# Therapeutic vulnerabilities exposed by the 9p21 loss identified through multiparametric drug screening inform rational combination strategies

**DOI:** 10.1038/s41698-026-01434-w

**Published:** 2026-04-18

**Authors:** Riccardo Bevilacqua, Paola Gasperini, Thomas Cantore, Catarina Macedo-Silva, Michael Pancher, Martina Radić, Haiyan Yue, Orsetta Quaini, Tarcisio Fedrizzi, Pamela Gatto, Roland Seiler, Bernhard Kiss, Valentina Adami, Francesca Lorenzin, Marianna Kruithof-de Julio, Bishoy Morris Faltas, Francesca Demichelis

**Affiliations:** 1 Department of Cellular, Computational and Integrative Biology, University of Trento, Trento, Italy; 2 Urology Research Laboratory, Department for BioMedical Research (DBMR), University of Bern, Bern, Switzerland; 3 Department of Urology, Hospital Centre Biel, Biel, Switzerland; 4 Department of Urology, Inselspital, Bern University Hospital, University of Bern, Bern, Switzerland; 5 Department for BioMedical Research (DBMR), Translational Organoid Resource (TOR), University of Bern, Bern, Switzerland; 6 Department of Medicine, Weill Cornell Medicine, New York, NY USA

**Keywords:** Cancer, Drug discovery, Oncology

## Abstract

Homozygous loss of the 9p21 locus encompassing CDKN2A, CDKN2B, and MTAP is the most frequent copy number alteration across tumor types, making it a promising target for precision medicine strategies. To explore drug vulnerabilities exposed by this loss, we generated 9p21 locus isogenic bladder cancer (BLCA) cell models to perform a multiparametric drug screen, testing 2,349 compounds. We identified cytarabine and methotrexate as significantly more effective in the 9p21 compromised BLCA cells. Analysis of morphological alterations further supported a genotype-specific activity of nucleoside analogs, nominating gemcitabine as a drug with greater efficacy in this context. To further exploit MTAP loss, we explored drug combinations targeting MTAP synthetic lethal partners, PRMT5 and MAT2A. Synergy between cytarabine and inhibitors of PRMT5 (MRTX1719) and MAT2A (AG-270) was mediated by a differential activation of DNA damage and replication stress markers, suggesting an exploitable vulnerability. In fact, rational drug combinations with ATR/CHK1 pathway inhibitors increased efficacy while maintaining 9p21-specificity. Finally, we confirmed the effectiveness of these combinations in cell models of pancreatic adenocarcinoma and pleural mesothelioma, two tumor types with high prevalence of MTAP loss and, most notably, in bladder cancer patient-derived organoids, underscoring the strong translational potential of our findings.

## Introduction

The 9p21 locus includes the tumor suppressor genes *CDKN2A* and *CDKN2B*, which encode key cell cycle regulators, and the metabolic gene *MTAP* (methylthioadenosine phosphorylase), involved in the salvage pathway for adenine and methionine, and co-deleted in about 80-90% of cancers with *CDKN2A* loss^1^. Using alternative exons, *CDKN2A* encodes for two unrelated proteins, p16^INK4a^ (p16) and p14^ARF^ (p14)^2,3^. P16 interacts with CDK4/6, thereby preventing RB phosphorylation and causing cell cycle arrest in the G1 phase of the cell cycle. On the other hand, p14 is involved in regulating the p53 pathway by directly inhibiting the MDM2 ubiquitin ligase, thereby stabilizing p53. *CDKN2B* encodes for another member of the INK4 family, p15^INK4b^ (p15), which is also responsible for the inhibition of CDK4/6 kinase activity^2^.

Overall, the locus harbors the most frequent copy number alteration (i.e., genomic deletion) across tumor types. Synthetic lethal (SL) vulnerabilities associated with 9p21 loss have been identified in cancer cell lines of different origins through loss-of-function screens^4–6^. Specifically, in cells lacking MTAP, 5-methylthioadenosine (MTA) accumulates and competes with the methyl donor S-adenosyltransferase (SAM) for binding to protein arginine methyltransferase 5 (PRMT5), leading to partial reduction of PRMT5 activity. This condition makes *MTAP*-deficient cells sensitive to further PRMT5 inhibition, either through direct targeting of PRMT5 or through the indirect inactivation of the primary SAM-producing enzyme, methionine adenosyltransferase 2A (MAT2A). To therapeutically exploit these synthetic lethal vulnerabilities, small molecule inhibitors targeting either the PRMT5-MTA complex (e.g., MRTX1719^7^ and AMG 193^8^) or MAT2A (e.g., AG-270 and IDE397^9^) have been developed, and are currently being tested in clinical trials (NCT05245500, NCT05094336, and NCT04794699).

Bladder cancer (BLCA) is the 9th most common cancer type in the world and a major cause of cancer-related death^10^. Most BLCAs are urothelial carcinomas that are classified as non-muscle invasive bladder cancer (NMIBC) or muscle-invasive bladder cancer (MIBC), with different prognoses and management^11^. Although antibody-drug conjugates, immunotherapy, and targeted therapy are offering new therapeutic opportunities, many patients do not respond to these treatments. In this scenario, advanced MIBC represents a major clinical challenge, characterized by extensive and dynamic clonal evolution that favors resistance to treatment, underscoring the need for new, effective therapies^12^ Several studies have investigated the molecular landscape of NMIBC and MIBC, revealing that *CDKN2A* loss at chromosome 9p21 is the most common copy number alteration in BLCA, occurring in approximately 42 and 22% of NMIBC and MIBC, respectively^13,14^. Therefore, precision medicine approaches for BLCA with 9p21 loss could be of interest to a wide range of patients, making bladder cancer a relevant context for investigating the functional and therapeutic implications of this deletion.

While the synthetic lethal interactions of cells with 9p21 loss have been widely studied using genetic-based approaches, we hypothesized that a multiparametric drug screening could expose new vulnerabilities of BLCA cells with 9p21 loss, and nominate drugs with a more direct translational outcome. Unlike cell viability screenings that provide only binary outcomes (live or dead), multiparametric drug screenings can capture complex cellular responses, offering insights into drug mechanisms of action, types of cell death, and molecular responses. The screening was performed in an isogenic pair of 9p21 wild-type (WT) and deficient BLCA cell lines. We found that 9p21-deficient BLCA cells are more sensitive than WT cells to the nucleoside analog cytarabine, because of higher levels of DNA damage and replication stress. To enhance the translational impact of our findings and maximize the efficacy and specificity of the compound, we tested rational drug combinations. Importantly, we provide evidence that cytarabine significantly combines with MAT2A and PRMT5 inhibitors in 9p21-deficient BLCA cell lines, with limited effects in WT cells, and that these treatments expose a cell vulnerability that can be specifically targeted by ATR/CHK1 pathway inhibitors. We validated our findings in tumor types characterized by a high frequency of MTAP deletion, such as mesothelioma and pancreatic adenocarcinoma, using cell line models. To establish their translational potential, we further confirmed these results in higher-complexity bladder cancer models based on patient-derived organoids (PDOs).

## Results

### A multiparametric drug screening identifies pharmacological vulnerabilities of bladder cancer cells with 9p21 loss

**Fig. 1:**
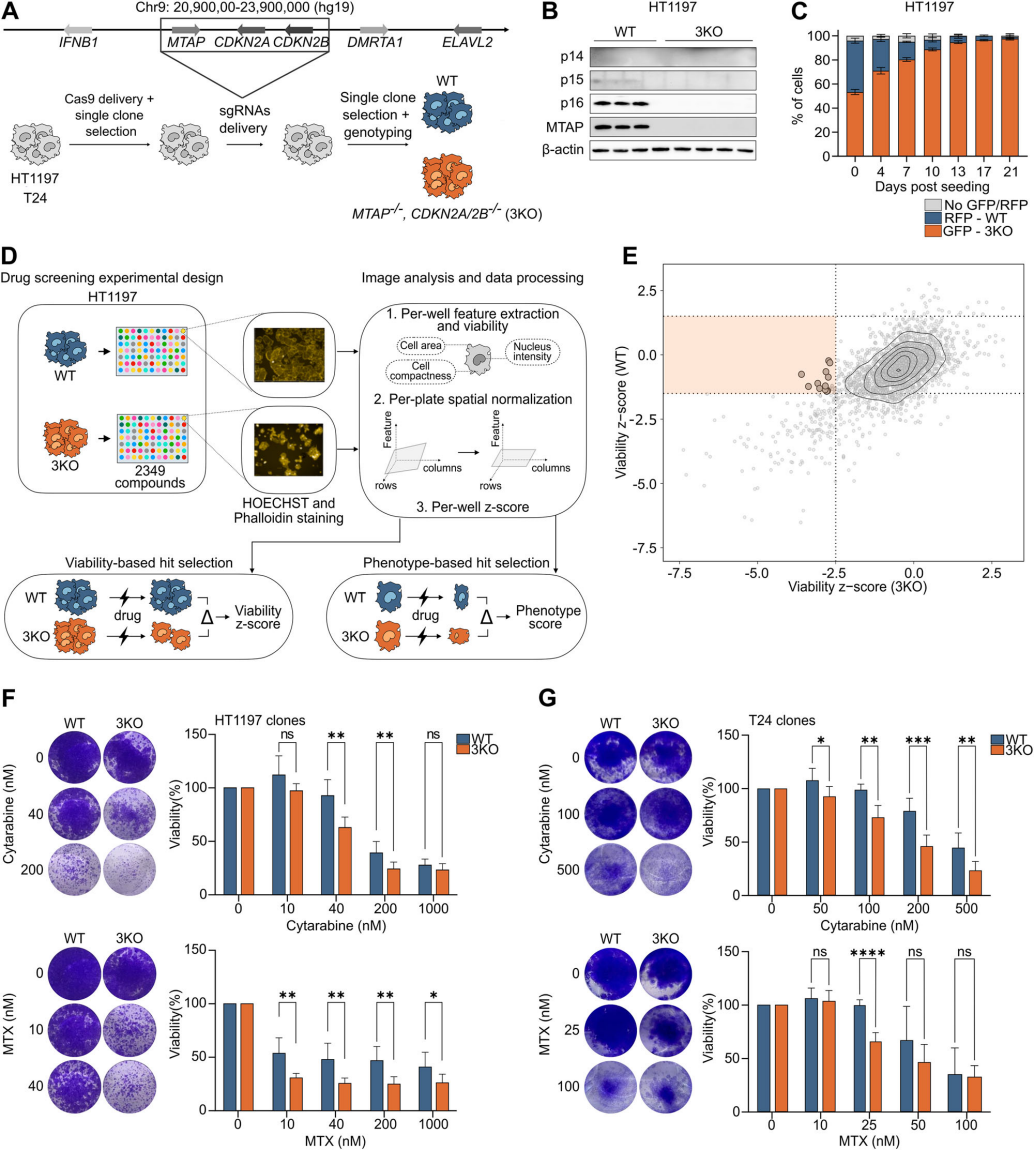
A multiparametric drug screening identifies pharmacological vulnerabilities of bladder cancer cells with 9p21 loss. **A** Schematic representation of the generation of 9p21 locus isogenic pairs. Several WT and 3KO clones were isolated for the HT1197 and T24 cell lines. **B** Western blot analysis of HT1197 WT and 3KO clones with the indicated antibodies. **C** Growth competition assays for HT1197 9p21 isogenic pair. WT (RFP-positive) and 3KO (GFP-positive) cells were co-cultured for 3 weeks, and the proportion of each cell population was measured every 3–4 days using Tali image-based cytometer (mean ± SD, *n* = 3). 3KO cells are more abundant from day 4 (*P* < 0.0001, unpaired t-test). **D** Schematic representation of the drug screening design performed in the HT1197 isogenic pair. After 48 h of treatment with a library of 2349 compounds, cells were fixed and stained with phalloidin and Hoechst. Fluorescent images were acquired and analyzed to calculate viability Z-scores and phenotype scores in WT and 3KO cells upon drug exposure. **E** Plot depicting the screening results. 18 drugs showed 3KO selective viability reduction (orange rectangle). Dashed lines indicate +1.5 and −1.5 viability z-score in WT cells and −2.5 viability z-score in 3KO cells. These thresholds were used to select drugs more effective for 3KO cells than WT. **F** Crystal violet survival assays performed with 2 HT1197 WT and 2 HT1197 3KO clones (mean ± SD, *n* = 6, 3 biological replicates per clone). Cells were treated with cytarabine and methotrexate (MTX) for 7 days. Representative crystal violet images of one WT and one 3KO clone are shown. *P* values were calculated by unpaired t-test. **G** Crystal violet survival assays performed with 2 T24 WT and 2 T24 3KO clones (mean ± SD, *n* = 6, 3 biological replicates per clone). Cells were treated with cytarabine and methotrexate (MTX) for 3 days. Representative crystal violet images of one WT and one 3KO clone are shown. *P* values were calculated by unpaired t-test. *P < 0.05; **P < 0.01; ***P < 0.001; ****P < 0.0001.

Our analysis of the TCGA MIBC cohort showed that approximately 26% of patients harbor homozygous deletion of *CDKN2A*/*CDKN2B*/*MTAP*, while only a minority (approximately 5%) harbor homozygous deletions of only *CDKN2A/2B*. Therefore, to create reliable cell models that mimic patient tumors with 9p21 loss, we analyzed the genomic alterations of a panel of BLCA cell lines derived from the DepMap data portal (https://depmap.org/portal, Cancer Cell Line Encyclopedia (CCLE) collection^15^) to identify cell lines with an intact 9p21 locus (WT) and WT RB1 status. The rationale for this selection was to evaluate the effects of *CDKN2A/2B* deletion on the cell cycle since these two genes encode for proteins that prevent RB1 phosphorylation via CDK4/6 interaction. We selected two of the six BLCA cell lines that satisfied these characteristics, HT1197 and T24, and deleted a region of approximately 210 kb encompassing *CDKN2A*, *CDKN2B*, and *MTAP* using the CRISPR/Cas9 technology to generate *CDKN2A*^−/−^/*CDKN2B*^−/−^/*MTAP*^−/−^ (3KO) cells. Isolation of single clones by limiting dilution was necessary to obtain cells with homozygous deletion (Fig. 1A) (see “Methods” for a detailed description of cell models generation). Control clones with a WT 9p21 locus were obtained by transducing HT1197 and T24 cells with a single-guide non-targeting RNA. Editing of the isolated clones was confirmed by PCR followed by Sanger sequencing, at the mRNA and protein level (Fig. 1B, Supplementary Fig. 1A). To finally test the specific contribution of *MTAP* vs *CDKN2A/2B*, we also generated HT1197 clones in which a 140 kb fragment encompassing only *CDKN2A/2B* (2KO clones) was deleted and that we employed in the validation steps (Supplementary Fig. 1A).

The isolated isogenic WT and 3KO clones were tested to assess the impact of the deleted genes. As *CDKN2A*/*2B* loss promotes unrestricted cell cycle progression and proliferation^2,16^, cell competition assays were performed by transducing WT and 3KO clones with lentiviral vectors carrying RFP and GFP fluorescent reporters (WT RFP-expressing cells and 3KO GFP-expressing cells) and were then mixed in a 50–50% ratio. We observed that HT1197 3KO clones proliferate faster and outcompete the WT cells after approximately 3 weeks of culture (*P* < 0.05, unpaired t-test) (Fig. 1C). On the other hand, the proportion of T24 3KO and WT clones remained similar over time (Supplementary Fig. 1B), possibly because T24 cells do not express p16^17^ (Supplementary Fig. 1A). Furthermore, we performed cell cycle analysis that demonstrated similar distribution of the cell cycle phases for both HT1197 and T24 WT and 3KO clones (Supplementary Fig. 1C) (*P* > 0.05, two-sided Wilcoxon test). The high proliferation rate of cancer cells harboring genomic alterations that compromise cell cycle checkpoints may limit the identification of cell cycle changes in our isogenic pairs at short time points (2 h).

*MTAP* deletion promotes the accumulation of MTA, leading to partial inhibition of PRMT5 activity, which is responsible for catalyzing the formation of symmetric dimethylarginine (SDMA) to target proteins^6^. Therefore, we measured SDMA in our models. As expected, 3KO clones of both cell lines have lower SDMA levels compared to WT clones, while no differences were observed between HT1197 WT and 2KO clones, consistent with partial impairment of PRMT5 specifically in 3KO clones (Supplementary Fig. 1D).

We selected the HT1197 9p21 isogenic pair (WT and 3KO) to perform a high-content drug screening with the aim of identifying selective pharmacological vulnerabilities exposed by this loss in bladder cancer cells. The use of an isogenic pair, WT and 3KO, with an identical genetic background, excludes inter-sample variability and ensures that observed drug response differences relate to the pharmacological intervention specific for the 9p21 loss. We screened a library of 2349 compounds at a single concentration of 1 µM for 48 h (Fig. 1D), which included anti-cancer small molecules in clinical use or part of clinical trials, and less characterized drugs not currently used for cancer treatment. Nuclei and actin filaments were stained following drug administration to determine the impact of the tested drugs on nuclei count as a proxy of cell viability and on cell morphology (i.e., cytoskeleton staining) (Supplementary Tables 5 and 6). Using nuclei count, we identified 18 compounds that preferentially reduced cell viability in 3KO cells (z-scores ≤ −2.5) and not in WT cells (−1.5 ≤ z-score ≤ 1.5) (Fig. 1E, Supplementary Fig. 1E). Among these drugs, the antifolate agent methotrexate (MTX) worked as a positive control based on Alhalabi et al.^17^, who recently demonstrated increased sensitivity of *MTAP*-deficient BLCA cells to the antifolate agent pemetrexed. We performed dose-response assays using HT1197 and T24 cells to validate the identified hits (Fig. 1F, G). To exclude that our results were influenced by clones’ selection, we tested the compounds in two isogenic clone pairs (WT and 3KO) for each cell line, obtaining consistent results. Crystal violet experiments confirmed significantly higher efficacy of MTX and cytarabine for the 3KO genotype (*P* < 0.05, unpaired t-test).

To understand whether deletion of the three genes was required to determine drug sensitivity, we assessed MTX and cytarabine activity in HT1197 2KO clones (Supplementary Fig. 1F). We observed that WT and 2KO clones were equally sensitive to the tested drugs, suggesting that *MTAP* deletion—but not *CDKN2A/2B* deletion—is required to increase sensitivity to the drugs (*P* > 0.05, unpaired t-test).

Collectively, these results confirmed that MTAP loss in bladder cancer cells confers sensitivity to the antifolate agent MTX, and identified the nucleoside analog cytarabine as a novel pharmacological vulnerability in this context.

### The phenotype-based hit selection further demonstrates sensitivity of cells with MTAP loss to nucleoside analogs

**Fig. 2:**
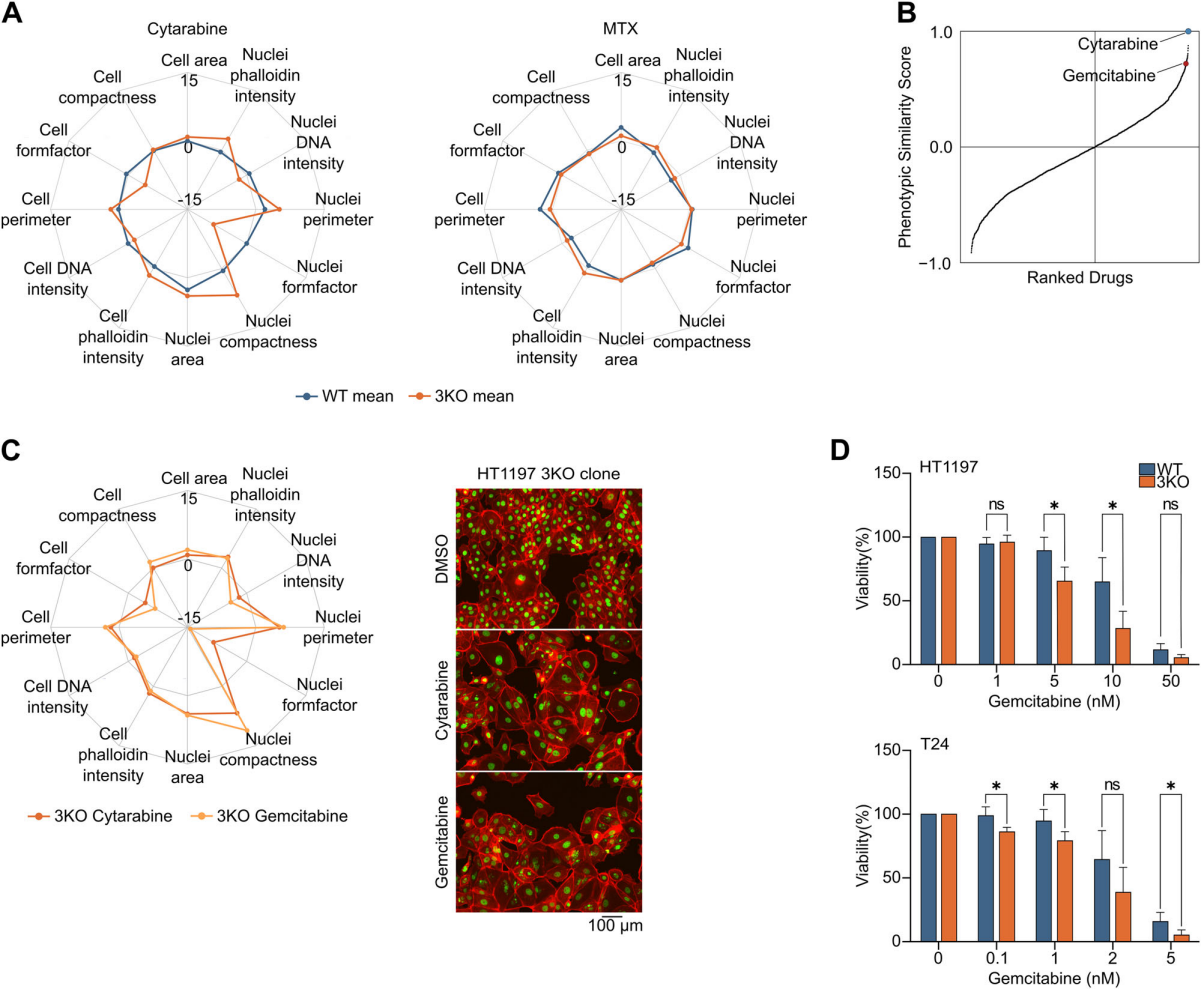
The phenotype-based hit selection potentially informs on drug sensitivity of cells with 9p21 loss. **A** Radar plots showing the phenotypic alterations induced by cytarabine and methotrexate in HT1197 WT and 3KO cells. **B** Correlation between the morphological changes induced by cytarabine and the drug screening compounds. Drugs were ranked according to the correlation score, and among the top hits, the pyrimidine analog gemcitabine is indicated. **C** Left, the radar plot shows the morphological alterations induced by cytarabine and gemcitabine in the 3KO clone. Right, fluorescent images showing the phenotypic alterations induced by cytarabine, gemcitabine, and DMSO (as control). Nuclei (green) and actin filaments (AF555 phalloidin, red) are stained. **D** CCK-8 survival assays performed with HT1197 and T24 WT and 3KO clones (mean ± SD, *n* = 3) treated with gemcitabine for 7 days (HT1197) or 3 (T24) days. *P* values were calculated by unpaired t-test. *P < 0.05.

Our drug screening tested 2349 drugs at a single dose and time point, possibly missing drugs that had a differential effect at a different concentration and/or timing. To maximize drug identification, we exploited the multiparametric feature of the screening and examined morphological alterations induced by drug treatment. For each object (i.e., cell or nucleus), 12 phenotypic features characterizing the area/shape, and intensity were analyzed, at the nucleus- and cell-level (area, compactness, formfactor, perimeter, DNA intensity, phalloidin intensity (see “Methods” for a detailed description of the analyses performed) (Supplementary Tables 6 and 7)). We focused on morphological alterations induced by cytarabine and MTX, reasoning that drugs with a similar mechanism of action will elicit a similar pattern of morphological changes. While MTX induced limited alterations of cell morphology in both genotypes (Fig. 2A), cytarabine had a stronger and 3KO-selective impact on cell morphology, particularly affecting nuclei-level features (Fig. 2A). We therefore looked for drugs that induced morphological changes similar to cytarabine by analyzing the correlation between the alterations induced by cytarabine and the other drugs in the screening. Interestingly, this analysis revealed that gemcitabine, a nucleoside analog similar to cytarabine and used as standard-of-care for the treatment of MIBC in combination with cisplatin^18^, scored among the top hits (Fig. 2B, C). Dose-response viability assays (performed in the range of 0.1–50 nM) demonstrated a significantly higher activity of gemcitabine in HT1197 and T24 3KO clones compared to WT cells, indicating that 3KO clones have increased sensitivity to different nucleoside analogs (Fig. 2D) (*P* < 0.05, unpaired t-test). These results provide evidence supporting the use of 9p21 genomic status as a biomarker to inform personalized treatment strategies involving nucleoside analogs.

### Cytarabine synergizes with PRMT5 and MAT2A inhibitors selectively in bladder cancer cells with MTAP loss

The use of drug combinations in cancer therapy can provide important advantages, including increased treatment efficacy, reduced toxicity, and limited development of drug resistance^19^. Therefore, in the context of a precision medicine approach, we explored potential drug combinations that would best exploit the vulnerabilities exposed by 9p21 loss (including MTAP deletion) in BLCA, utilizing already known drugs and drugs identified by our screening. Specifically, we tested whether cytarabine, gemcitabine, and MTX could be combined with small-molecule inhibitors of the synthetic lethal partners of *MTAP*, MAT2A, and PRMT5^7,20^ to enhance cell death and increase genotype specificity in 3KO cells. First, we investigated the activity of the MAT2A inhibitor AG-270 and the PRMT5 inhibitor MRTX1719, which selectively binds to PRMT5 complexed with MTA, to define the ideal dose for a clear genotype-specific effect when administered alone in our cell models. Cell viability assays demonstrated significantly higher sensitivity of 3KO cells to AG-270 and MRTX1719 compared to WT clones (Supplementary Fig. 3A) (*P* < 0.05, unpaired t-test). As control, we also tested AG-270 and MRTX1719 in HT1197 WT and 2KO clones, which maintain MTAP expression, demonstrating no differential sensitivity to the treatments (Supplementary Fig. 3B).

**Fig. 3:**
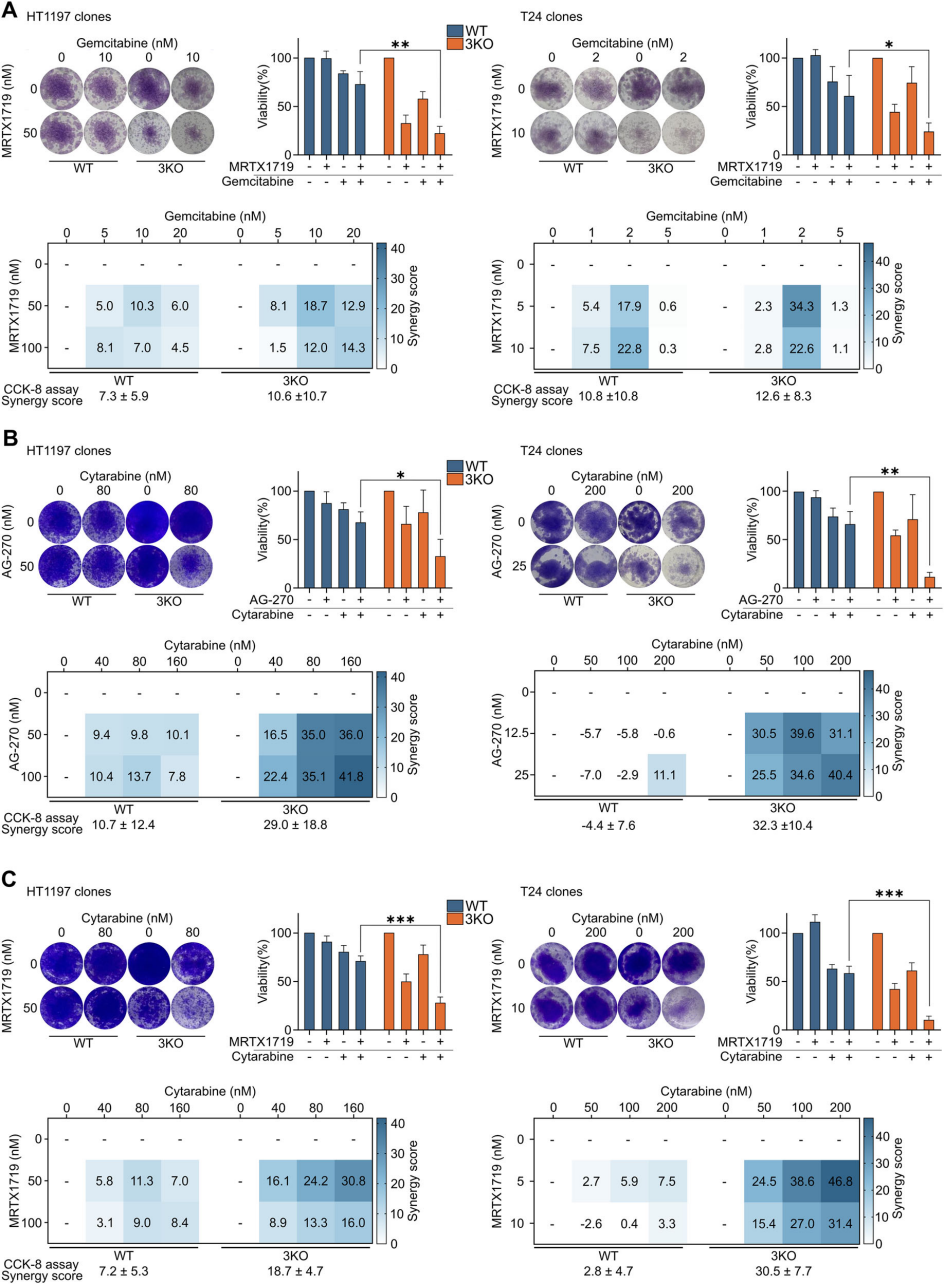
Cytarabine synergizes with AG-270 and MRTX1719 to induce cell death in clones with 9p21 deletion. **A** HT1197 and T24 clones were treated with MRTX1719 for 2 days, followed by combined MRTX1719 and gemcitabine treatment for 3 (T24 clones) or 5 (HT1197 clones) days. Cell viability was measured by crystal violet and CCK-8 assays. Crystal violet experiments and barplots (top) show representative images of selected gemcitabine and MRTX1719 combinations, and of CCK-8 measured cell viability, respectively. Dose-response matrices (bottom) show synergy scores calculated between gemcitabine and MRTX1719 at the tested concentrations specifically in HT1197 (left) and T24 (right) 3KO cells (*n* = 3). Synergy was calculated with two models (HSA and Bliss) using SynergyFinder 3.1, and HSA synergy scores and summary synergy scores with 95% confidence interval for the CCK-8 experiments are shown. *P* values were calculated by unpaired t-test. **B** Cells were treated with AG-270 for 2 (T24 clones) or 6 (HT1197 clones) days, followed by combined AG-270 and cytarabine treatment for 3 (T24 clones) or 7 (HT1197 clones) days. Cell viability and synergy scores were measured and calculated as in (**A**). Crystal violet experiments and barplots (top) show representative images of selected cytarabine and AG-270 combinations, and of CCK-8 measured cell viability, respectively. Dose-response matrices (bottom) show synergy scores between cytarabine and AG-270 at the tested concentrations, specifically in HT1197 (left) and T24 (right) 3KO cells (*n* = 3). *P* values were calculated by unpaired t-test. **C** HT1197 and T24 clones were treated with MRTX1719 for 2 days, followed by combined MRTX1719 and cytarabine treatment for 3 (T24 clones) or 5 (HT1197 clones) days. Cell viability and synergy scores were measured and calculated as in (**A**). Crystal violet experiments and barplots (bottom) show representative images of selected cytarabine and MRTX1719 combinations, and of CCK-8 measured cell viability, respectively. Dose-response matrices (bottom) show synergy scores between cytarabine and MRTX1719 at the tested concentrations specifically in HT1197 (left) and T24 (right) 3KO cells (*n* = 3). *P* values were calculated by unpaired t-test. *P < 0.05; **P < 0.01; ***P < 0.001.

Since AG-270 and MRTX1719 target an epigenetic pathway (i.e., PRMT5-mediated symmetric di-methylation of arginine marks), it has been reported that several days of treatment are required to observe a potent anti-tumor activity^7^. Therefore, we reasoned that a sequential administration protocol with AG-270 or MRTX1719 pre-treatment followed by chemotherapy was preferential to reduce toxicity mediated by long treatment with cytarabine, gemcitabine, or MTX. When administered in combination with MTX, MRTX1719 did not display any synergistic interaction for both HT1197 and T24 3KO clones at the concentrations tested (synergy score < 10) (Supplementary Fig. 3C). Combination of MRTX1719 with gemcitabine showed synergy at only a few concentrations tested and showed limited specificity for the 3KO clones compared to the WT ones in both cell lines (Fig. 3A). On the other hand, cell viability assays in HT1197 and T24 clones showed synergy of AG-270 and MRTX1719 in 3KO clones when combined with cytarabine (Fig. 3B, C). Indeed, Highest Single Agent (HSA) model demonstrated a synergistic interaction between cytarabine and AG-270 or MRTX1719 (synergy score >10)^21^. Importantly, synergy was observed only in 3KO clones for both cell lines but not in WT or 2KO clones (Supplementary Fig. 3D), strongly supporting the selectivity of the treatments in bladder cancer cells with MTAP loss.

### Combination of cytarabine with AG-270 or MRTX1719 triggers DNA damage, replication stress, and apoptosis selectively in MTAP-deleted cells

**Fig. 4:**
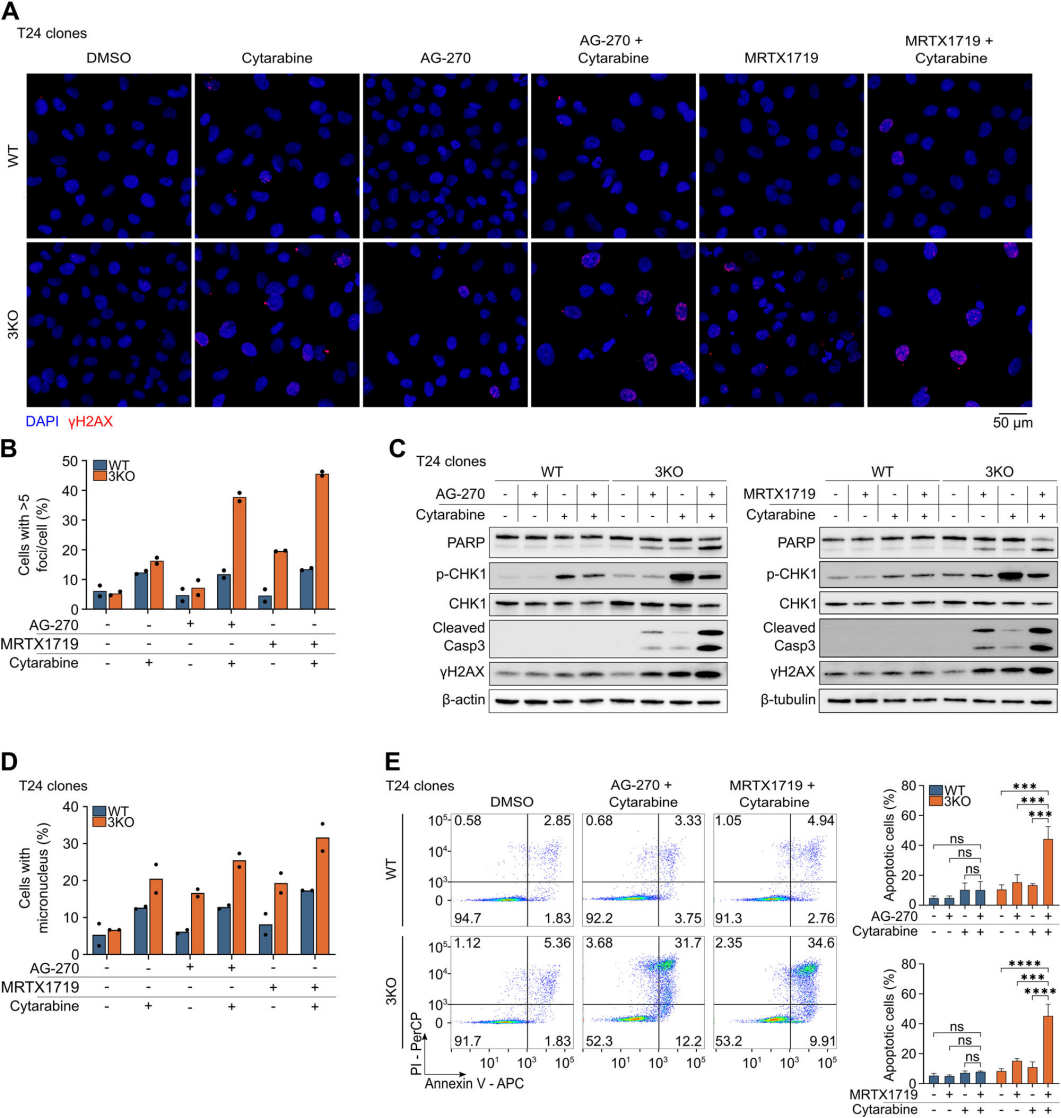
Biological effects of cytarabine and its combination with PRMT5 and MAT2A inhibitors in T24 WT and 3KO clones. **A** Representative images of γH2AX foci staining in T24 WT and 3KO cells treated with the indicated drugs. Cells were treated with DMSO (as control), AG-270 (25 nM), or MRTX1719 (10 nM) for 2 days, followed by combination with cytarabine (200 nM) for 3 days. **B** γH2AX foci quantification in T24 WT and 3KO cells treated with the indicated drugs (*n* = 2). Cells were treated as in (**A**). Data represents the proportion of cells with >5 foci/nucleus. **C** Western blot analysis of the indicated proteins in T24 WT and 3KO cells treated with DMSO (as control), AG-270, or MRTX1719. T24 cells were treated as in (**A**). **D** Quantification of micronucleus-positive cells in T24 WT and 3KO cells treated with the indicated drugs (n = 2). Cells were treated as in (**A**). **E** Representative FACS profiles (left) and quantification (right) of Annexin V - PI staining for T24 9p21 isogenic pair treated with the indicated drugs, alone or in combinations (mean ± SD, *n* = 3). T24 cells were treated as in (**A**). *P* values were calculated by one-way ANOVA. ***P < 0.001; ****P < 0.0001.

Cytarabine, AG-270, and MRTX1719 are known to cause DNA damage in cells. Cytarabine competes with cytidine for DNA incorporation, causing termination of DNA chain elongation^22^, while the inhibition of PRMT5 activity triggers the accumulation of R-loops, which leads to DNA damage^20^. Therefore, in order to investigate how cytarabine, AG-270, and MRTX1719 act synergistically in our models, we evaluated their single and combined effects on DNA damage. Measurement of H2AX phosphorylation (γH2AX) in immunofluorescence (IF) demonstrated a higher induction of double-strand breaks (DSBs) in T24 and HT1197 3KO clones compared to WT cells in response to treatments (Fig. 4A, B and Supplementary Fig. 4A). While the monotherapies caused limited DSBs formation, drug combinations induced a strong increase in γH2AX foci formation, which was more evident for T24 cells. Western blot analysis of T24 and HT1197 clones confirmed increased levels of γH2AX in response to all treatment conditions only for 3KO clones, which were particularly marked in the combination experiments (Fig. 4C and Supplementary Fig. 4B).

Given that cytarabine incorporation into DNA causes a stall of replication fork progression^23^, we checked by western blot the levels of CHK1 phosphorylation (p-S345 CHK1), a marker of replication stress^24^. 3KO clones showed increased levels of p-CHK1 when treated with cytarabine alone or in combination with PRMT5 and MAT2A inhibitors compared to WT cells (Fig. 4C and Supplementary Fig. 4B), indicating induction of replication stress by drug treatment selectively in cells with 9p21 loss.

In addition, we observed formation of micronuclei in response to treatments, suggesting that drugs, and particularly drug combinations, caused genomic instability in 3KO cells (Fig. 4D). Interestingly, we noticed the presence of γH2AX-positive micronuclei, which has been shown to be favored by agents that cause replication stress^25^, further supporting replication stress induction in 3KO cells.

Finally, Annexin V – propidium iodide (PI) staining demonstrated a significant increase in the percentage of apoptotic cells following the combination of AG-270 and MRTX1719 with cytarabine, selectively in 3KO cells for T24 (*P* < 0.001, one-way ANOVA) and, in part, for HT1197 cells (Fig. 4E, Supplementary Fig. 4C) (*P* < 0.05, one-way ANOVA). Apoptosis was further evaluated by measuring the levels of cleaved-caspase 3 and of PARP cleavage in response to the treatments (Fig. 4C and Supplementary Fig. 4B). Consistent with Annexin V–PI analysis, the induction of these apoptotic markers was limited to 3KO cells and increased when the drugs were combined.

Collectively, these data demonstrate that the effect on cell viability mediated by cytarabine and its combinations with AG-270 and MRTX1719 (see Fig. 3B, C) preferentially affects MTAP-deleted cells by triggering higher levels of DNA damage, replication stress, and apoptosis. These results point to DNA damage and replication stress as new pathways vulnerabilities exposed by the administrations of cytarabine with AG-270 or cytarabine with MRTX1719 in MTAP-deleted cells.

### Replication stress induced by cytarabine, AG-270, and MRTX1719 sensitizes 9p21-deleted cells to the ATR inhibitor VX970

**Fig. 5:**
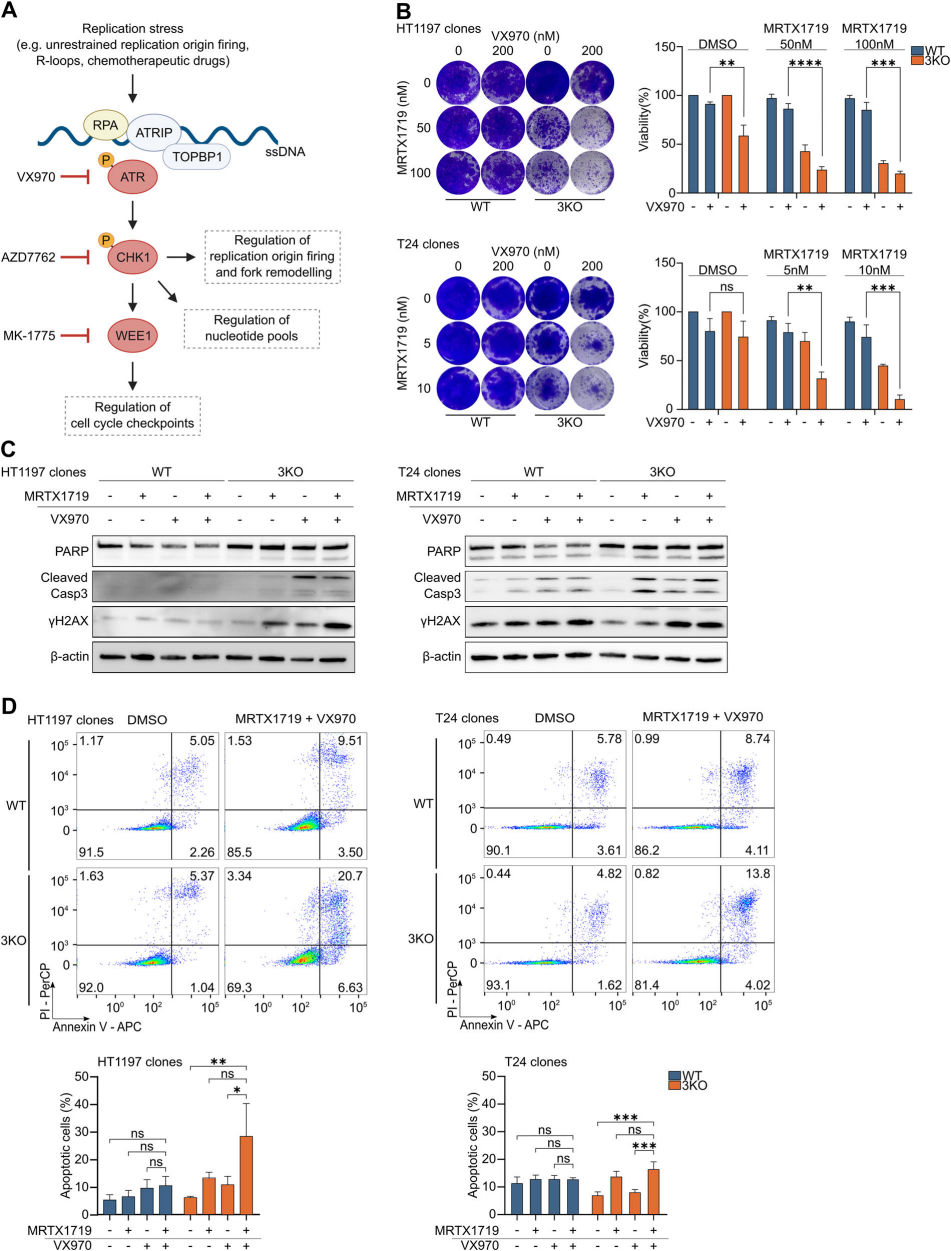
The ATR inhibitor VX970 combines with cytarabine and MRTX1719 to increase cell death in cells with 9p21 loss. **A** Schematic representation of the ATR-CHK1 pathway and of its biological functions. Selective inhibitors acting at different pathway level are indicated. ATRIP ATR-interacting protein, RPA replication protein A, ssDNA single-stranded DNA, TOPBP1 DNA topoisomerase 2-binding protein. **B** Crystal violet survival assays (left) and CCK-8 assays (right) of HT1197 and T24 cells treated with MRTX1719 in combination with VX970 (mean ± SD, *n* = 3). T24 cells were treated with DMSO (as control) or MRTX1719 for 2 days, followed by combination with VX970 for 4 days. HT1197 cells were treated with DMSO (as control) or MRTX1719 for 2 days, followed by combination with VX970 for 5 days. *P* values were calculated by unpaired t-test. **C** Western blot analysis of the indicated proteins in HT1197 and T24 cells in WT and 3KO cells treated with DMSO, MRTX1719, or VX970 as described in (**B**). **D** FACS analysis quantification of Annexin V - PI staining for the HT1197 and T24 clones treated with DMSO, MRTX1719, or VX970 as described in (**B**) (mean ± SD, *n* = 3). Replicates of the FACS experiments for HT1197 cells were conducted concurrently as the experiments in Supplementary Figs. 4D and 5F, and share the same controls. *P* values were calculated by one-way ANOVA. *P < 0.05; **P < 0.01; ***P < 0.001; ****P < 0.0001.

We reasoned that DNA damage and replication stress preferentially triggered in MTAP-deleted cells by the administrations of cytarabine with AG-270 or with MRTX1719 could be further exploited for treatment strategies by targeting ATR-CHK1 pathway, activated in response to replication stress to preserve genome integrity during DNA replication. Previous studies have demonstrated that PRMT5 inhibition in *MTAP*-deficient cells leads to the accumulation of R-loops^20^, possibly because of reduced PRMT5-mediated methylation of DDX5, a crucial helicase for R-loops resolution^26^, resulting in replication stress due to collisions between the replication and transcription machineries. Additionally, cytarabine induced replication stress selectively in 3KO cells as shown by increased p-CHK1 levels by western blot analysis (Fig. 4C and Supplementary Fig. 4B) and the levels of p-CHK1 are consistently higher—at least for T24 cells—in 3KO compared to WT cells, even in untreated condition, suggesting that deletion of the 9p21 locus could cause an increase in basal replication stress (Fig. 4C). Based on these observations, we reasoned that cytarabine and PRMT5 inhibition, alone and in combination, could increase the dependency of 3KO cells on the ATR/CHK1 pathway, which is central in the cellular response to replication stress. The ATR/CHK1 pathway induces cell cycle arrest in S-G2 phase and prevents mitotic entry of the cells with excessive DNA damage, thereby limiting genomic instability^27^ (Fig. 5A). First, we checked our drug screen for compounds targeting the ATR-CHK1 pathway, and we found AZD7762 and MK-1775, known inhibitors of CHK1 and WEE1. We observed a trend for higher sensitivity of the HT1197 3KO clone compared to the WT clone to both drugs (AZD7762 WT z-score = −3.60, 3KO = −5.06; MK-1775 WT z-score = −1.12, 3KO = −2.06) (Supplementary Fig. 5A), although the two drugs were excluded from our hit prioritization since their viability z-scores were out of the considered range (i.e., z-scores ≤ −2.5 for 3KO and −1.5 ≤ z-score ≤ 1.5 for WT). These screening results further support our hypothesis that cells with 9p21 loss could be more prone to accumulate additional DNA damage in response to inhibition of the ATR/CHK1 pathway. Therefore, our strategy was to inhibit ATR as it acts upstream in the signaling pathway and has a broader function in the replication stress response than CHK1 and WEE1^28^. We selected the ATR inhibitor VX970, which was absent from the tested libraries, since several clinical trials are evaluating its activity alone and in combination with chemotherapy or radiotherapy for cancer treatment^29^. We performed cell viability assays administering cytarabine, MRTX1719, and AG-270 in combination with VX970. Similar to cytarabine combination experiments, we used a sequential drug administration protocol. We observed that VX970 combined with MRTX1719 or AG-270 significantly reduced cell viability selectively in HT1197 and T24 3KO cells (Fig. 5B and Supplementary Fig. 5B) (*P* < 0.01, unpaired t-test). A similar effect was observed for the combination of the CHK1 inhibitor AZD7762 with MRTX1719 in T24 3KO cells (Supplementary Fig. 5C). In addition, we observed increased sensitivity of HT1197 3KO cells to the combination of cytarabine and VX970 compared to WT cells (Supplementary Fig. 5D).

Next, we evaluated the induction of DNA damage and apoptosis by VX970 in combination with MRTX1719 or cytarabine. Immunoblot analyses demonstrated higher DNA damage (γH2AX levels) in 3KO clones in response to drug combinations (Fig. 5C and Supplementary Fig. 5E). Annexin V – PI staining showed induction of apoptosis in HT1197 3KO clones following MRTX1719 treatment alone, which is enhanced by co-treatment with VX970 in HT1197 cells (*p* value = 0.0588) (Fig. 5D). On the other hand, the Annexin V – PI staining for T24 cells treated with MRTX1719 and VX970 showed only a modest increase in the percentage of apoptotic cells compared to cells treated with monotherapies (Fig. 5D). Similar results were obtained by immunoblot detection of cleaved-caspase 3 and of cleaved PARP levels in response to treatments (Fig. 5C).

Taken together, these results suggest that cytarabine, MAT2A, and PRMT5 inhibitors can be effectively combined with ATR inhibition to increase cell death in cells with MTAP loss.

### Combination of cytarabine and VX970 with MRTX1719 is effective in different cancer types with *MTAP* deficiency

**Fig. 6:**
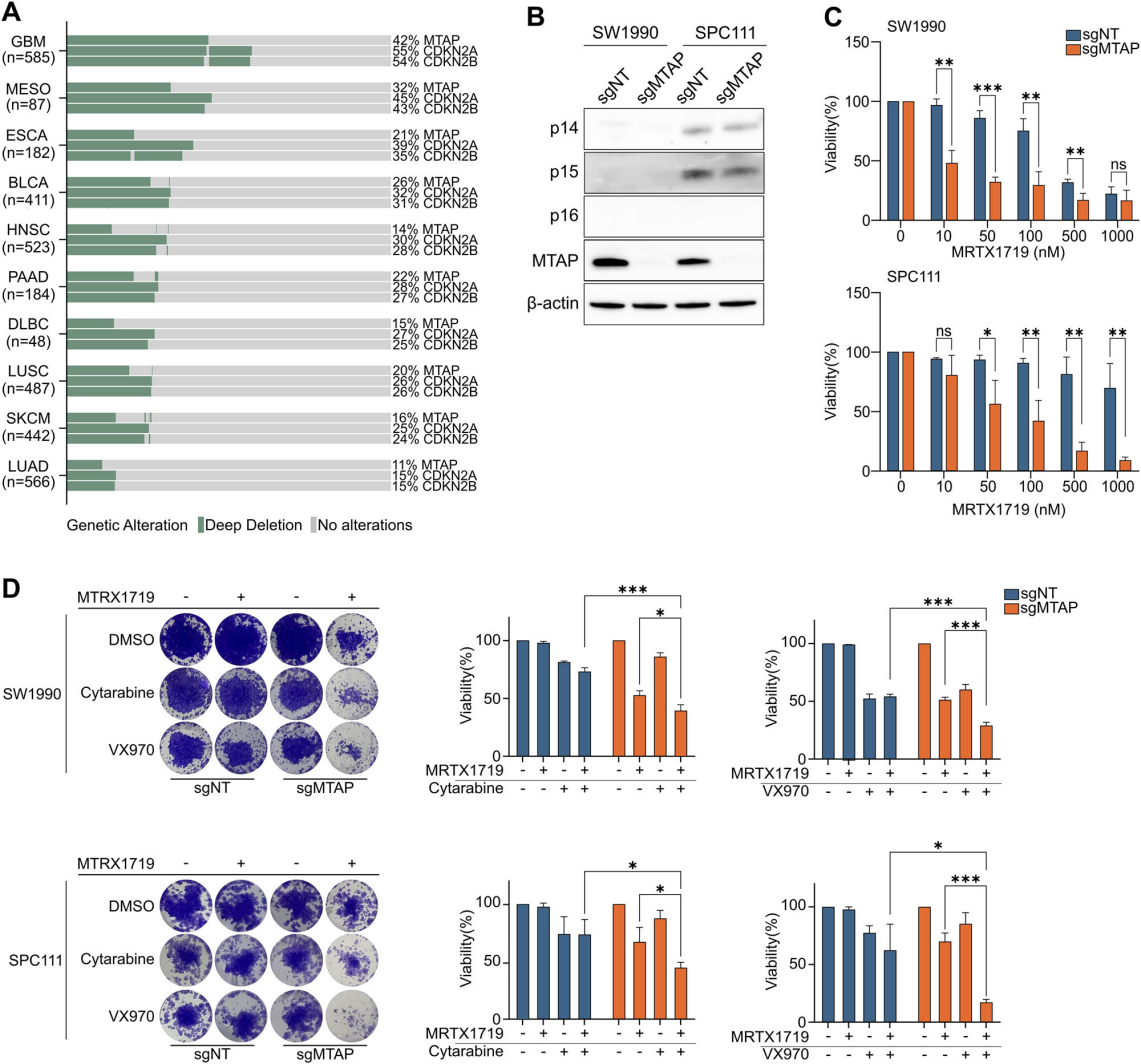
Combination of MRTX1719 with cytarabine and VX970 in pancreatic adenocarcinoma and mesothelioma cellular models. **A** Oncoprint showing the frequency of *CDKN2A*, *CDKN2B*, and *MTAP* homozygous deletion from the TCGA pan-cancer atlas 2018^45^. The ten tumor types with the highest frequency of *CDKN2A* deletion are shown. GMB glioblastoma, MESO mesothelioma, ESCA esophageal carcinoma, BLCA bladder cancer, HNSC head and neck squamous cell carcinoma, SKCM skin cutaneous melanoma, PAAD pancreatic adenocarcinoma, DLBC lymphoid neoplasm diffuse large B-cell lymphoma, LUSC lung squamous cell carcinoma, LUAD lung adenocarcinoma. **B** Western blot analysis of SW1990 and SPC111 WT (sgNT) and MTAP KO (sgMTAP) cell lines with the indicated antibodies. **C** CCK-8 assay quantification of SW1990 and SPC111 WT and KO cells treated with DMSO (as control) and MRTX1719 for 7 days. *P* values were calculated by unpaired t-test. **D** Crystal violet survival assays (left) and CCK-8 assays (right) of SW1990 and SPC111 cells treated with MRTX1719 in combination with cytarabine or VX970 (mean ± SD, *n* = 3). Cells were treated with DMSO (as control), or MRTX1719 for 2 days, followed by combination with cytarabine or VX970 for 5 days. *P* values were calculated by unpaired t-test. *P < 0.5; **P < 0.01; ***P < 0.001.

To expand the therapeutic applicability of the identified MTAP loss selective drug combinations in different cancer types, we evaluated their activity in pleural mesothelioma and pancreatic adenocarcinoma cellular models that have a frequency of *MTAP* loss of 32% and 22%, respectively (Fig. 6A). Moreover, both pleural mesothelioma and pancreatic adenocarcinoma have poor prognosis and are in urgent need of new therapeutic options^30,31^.

Since we previously showed that increased drug sensitivity to cytarabine and MRTX1719 is mainly defined by the *MTAP* status, we selected one pleural mesothelioma cell line, SPC111 and one pancreatic adenocarcinoma cell line, SW1990 that had a WT *MTAP* status (Supplementary Fig. 6A) and transduced these cell lines with a lentiviral vector to induce *MTAP* KO (Fig. 6B and Supplementary Fig. 6B). To evaluate synthetic lethality, we treated SW1990 and SPC111 cells with MRTX1719, confirming higher sensitivity of *MTAP* deficient cells to PRMT5 inhibition (Fig. 6C). Next, we treated SW1990 and SPC111 cells with MRTX1719 in combination with cytarabine or VX970. We found that both drug combinations were significantly more effective than single treatments in a genotype-specific manner, with limited toxicity for WT cells (Fig. 6D) (*P* < 0.05, unpaired t-test).

Overall, these results further support the efficacy and selectivity of PRMT5 inhibition in combination with cytarabine and VX970 for cells with MTAP loss and suggest that, besides BLCA, other tumor types may benefit from these treatments.

### Combination of cytarabine and VX970 with MRTX1719 is effective in BLCA patient-derived organoids (PDOs) with *MTAP* deficiency

**Fig. 7:**
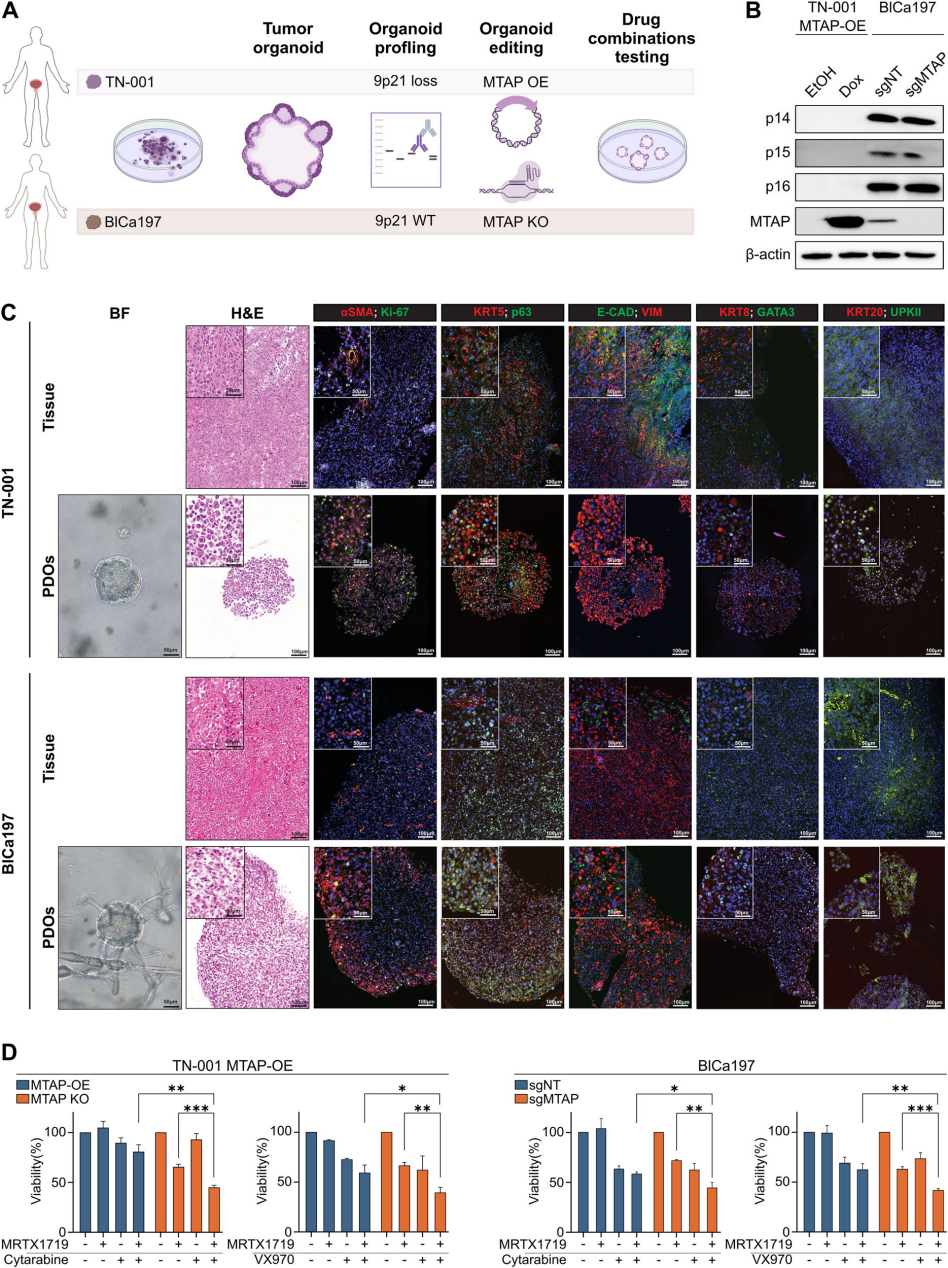
Human BLCA PDOs edited for MTAP expression show differential activity upon MRTX1719 in combination with cytarabine and VX970. **A** Schematic illustration of the workflow for PDOs generation, editing (MTAP inducible overexpression (OE) or MTAP KO), and testing of the drug combinations. **B** Western blot analysis of TN-001 with and without MTAP overexpression (OE) and BlCa197 (WT-sgNT and MTAP KO-/sgMTAP) PDOs with the indicated antibodies. TN-001 were treated with doxycycline (Dox) 1 µg/ml to induce MTAP overexpression or ethanol (EtOH) as control. **C** Expression of a panel of classical BLCA markers by immunofluorescence in the PDOs and the corresponding parental tissue of both TN-001 and BlCa197 samples: Brightfield (BF), Hematoxylin and Eosin (H&E) staining images and multiplexed immunofluorescence staining of αSMA (red), Ki-67 (green); KRT5 (red), p63 (green); E-CAD (green), VIM (red); KRT8 (red), GATA3 (green); KRT20 (red) and UPKII (green). DAPI dye (Blue) represents the nucleus location. **D** Cell viability assays (CellTiter-Glo 3D) of TN-001 and BlCa197 PDOs treated with MRTX1719 in combination with cytarabine or VX970 (mean ± SD, *n* = 3). Cells were treated with DMSO (as control), or MRTX1719 for 2 days, followed by combination with cytarabine or VX970 for 5 days. *P* values were calculated by unpaired t-test. *P < 0.5; **P < 0.01; ***P < 0.001.

PDOs are considered more physiological human cancer models than cancer cell lines because they retain key phenotypic and histological features of the corresponding parental tissue and can predict treatment responses in cancer patients^32,33^. Therefore, to evaluate the translational potential of our findings, we tested the efficacy and genotype selectivity of the drug combinations in two MIBC PDO-lines (derived from TN-001 and BlCa197 patients) that exhibit sustained in vitro proliferation while retaining patient‑specific genomic and phenotypic features. These two PDO‑lines were chosen because they exhibit distinct MTAP statuses, enabling us to test whether the observed drug‑combination effects are consistent across biologically divergent contexts (Fig. 7A). Specifically, TN-001 sample harbors a deletion of the locus, with no expression of p14, p15, p16, and MTAP, while BlCa197 has a 9p21 WT status with expression of the 4 proteins (Fig. 7B). Therefore, we transduced TN-001 cells with a lentiviral vector encoding for *MTAP* under the control of a doxycycline-inducible promoter to overexpress MTAP, and BlCa197 with a lentiviral vector to induce *MTAP* KO (Fig. 7B). To also verify that PDOs recapitulate key aspects of the parental tissue composition, we tested the expression of a panel of classical BLCA markers by immunofluorescence. Both pairs of PDO and parental tissue express hallmarks of BLCA biology, predominantly dominated by the epithelial/mesenchymal state, while maintaining their characteristic heterogeneity (Fig. 7C). Specifically, we observed the coexistence of both basal—cytokeratin 5 (KRT5) and p63—and luminal—cytokeratin 8 (KRT8), GATA3 and uroplakin II (UPKII)—epithelial differentiation markers, as well as E-cadherin (E-CAD), although less expressed in the BlCa197 sample. Cytokeratin 20 (KRT20) was instead absent or only residual in both samples. Additional mesenchymal markers, alpha smooth muscle actin (αSMA) and vimentin (VIM), were included for phenotypic characterization, highlighting this mixed histology^34^. The presence of Ki-67^+^ cells demonstrated the proliferative state of PDOs.

We then tested the response of PDOs to our drug combinations. As expected, MTAP overexpression in TN-001 organoids rescues MRTX1719 activity, while *MTAP* KO selectively increases MRTX1719 activity in BlCa197 (Supplementary Fig. 7A). We finally evaluated the efficacy of the drug combinations, MRTX1719 with cytarabine and MRTX1719 with VX970 on the PDO lines, demonstrating that the combinations were significantly more effective than the administration of single drugs for both models and that, this effect was more pronounced in *MTAP*-deficient PDOs compared to *MTAP*-proficient PDOs (Fig. 7D).

Overall, these data suggest that the observed drug‑combination effects are consistent across BLCA models with higher clinical relevance, supporting the robustness and relevance of the findings.

## Discussion

Highly recurrent genomic alterations are of considerable interest in translational research due to their potential clinical relevance. Such alterations may serve as prognostic biomarkers, inform on therapeutic response or patient stratification, or can be systematically explored for the exposure of new actionable vulnerabilities through diverse screening approaches, including genetic, pharmacological, and functional assays. The frequency across tumor types of the 9p21 locus genomic deletion is an iconic example.

In this study, we explored the therapeutic vulnerabilities associated with 9p21 loss in BLCA. Co-occurrence of *CDKN2A/2B* and *MTAP* deletions interests 26% of TCGA MIBC patients (*p* value < 0.001 based on cBioPortal two-sided Fisher Exact Test), while deletions affecting *CDKN2A/2B* are comparatively infrequent (5.6%), and *CDKN2A* focal loss is rare (0.5%). While SL interactions in cells with this impairment have been widely studied using genetic approaches, the pharmacological exploitation of these interactions remains understudied.

Leveraging an isogenic model of 9p21 WT and knockout BLCA cells, we performed a multiparametric high-content drug screen to identify compounds selectively active in 9p21-deficient contexts. Our screen uncovered an increased sensitivity of 9p21-deficient cells to cytarabine, a nucleoside analog not previously associated with 9p21 loss in BLCA.

To untangle the contribution of MTAP and the cell-cycle-related genes encoded by *CDKN2A/2B*, we tested the hits nominated by the drug screening (performed on one isogenic pair, WT vs 3KO clones) on several 3KO clones and on 2KO clones (*CDKN2A*^−/−^/*CDKN2B*^−/−^). The same approach was applied for all drugs and drug combinations validated throughout our study. First, we tested cytarabine and MTX in 2KO clones, observing no differential response to the drugs compared to WT cells (Supplementary Fig. 1F). Second, we tested AG-270 (MAT2Ai) and MRTX1719 (PRMT5i), demonstrating resistance to the treatments (Supplementary Fig. 2B). Third, we studied the response of 2KO cells to combinations of cytarabine with AG-270 or MRTX1719, confirming no activity of the combinations in the genotype (Supplementary Fig. 2D). All these experiments point to the loss of MTAP as responsible for the sensitivity of the 3KO cells to cytarabine, AG-270, MRTX1719 and their combinations. We cannot exclude that the lack of pharmacological vulnerabilities directly ascribable to *CDKN2A/2B* loss as a result of the drug screening is influenced by the limited expression of the proteins encoded by *CDKN2A/2B* in HT1197 model that expresses p16, but not detectable levels of p14 and p15.

Aware that a primary limitation of our screening approach is that all compounds were tested, in replicate, at a fixed concentration (of 1 μM), therefore likely missing compounds with genotype-specific response at different concentrations, we exploited a phenotype-based analysis of cell morphology to select drugs that produced similar patterns of morphological changes, which were possibly driven by a mechanism of action similar to that of the drug of interest. This approach identified gemcitabine as a second nucleoside analog with enhanced activity in 3KO cells.

Gemcitabine—a chemotherapeutic drug standard of care for BLCA treatment—is an analog of cytarabine with unique properties, including the inhibition of the ribonucleotide reductase, the cytidine triphosphate synthetase, and the deoxycytidylate deaminase that contribute to its activity in cancer cells^35^. In our settings, dose-response viability assays demonstrated higher gemcitabine activity in HT1197 and T24 3KO cells, but with a reduced genotype selectivity compared to cytarabine. These findings are consistent with the analysis of Cancer Dependency Map (DepMap) data, which shows that BLCA cells with *CDKN2A* deletion are more sensitive to gemcitabine than WT cells^36^. On the other hand, Alhalabi et al. observed a similar response of *MTAP*-proficient and -deficient BLCA cells to gemcitabine treatment, possibly due to higher drug concentrations^17^.

Despite the higher efficacy in 9p21 aberrant BLCA cells, cytarabine and gemcitabine have, as expected, a clear effect on the viability of 9p21 WT cells. To improve the specificity and efficacy of treatment, we tested drug combinations based on the known synthetic lethal interactions of *MTAP*, leveraging newly developed PRMT5 and MAT2A inhibitors (MRTX1719 and AG-270, respectively). We show that the combination of cytarabine with MRTX1719 or AG-270 exhibits synergistic and selective cytotoxicity in *MTAP*-deficient models, while, in our experimental setting (e.g., models, treatment schedules), gemcitabine did not exhibit synergy but was significantly more effective than single treatments only for a limited range of concentrations. The latter diverges from the literature^37^, possibly due to different settings.

Our mechanistic studies revealed that cytarabine and inhibitors of PRMT5 and MAT2A induce DNA damage and replication stress in cells with 9p21 loss, suggesting dependency of 3KO cells on the activity of the ATR/CHK1 pathway that is central in the cellular response to replication stress, and highlighting replication stress response inhibition as an additional actionable vulnerability. Replication stress is considered a biomarker of sensitivity to ATR inhibitors^38^ and possibly to CHK1 inhibitors in *CDKN2A*-deleted head and neck cancer^39^. Here we demonstrated that rational drug combinations based on inhibitors of the ATR/CHK1 pathway significantly increase cell death in 3KO cells that were pre-treated with cytarabine, AG-270, and MRTX1719. Interestingly, while synergism between ATR inhibition and cytarabine has been reported in several tumor types^40,41^, the correlation to 9p21 status has never been reported before. We speculate that ATR inhibition prevents 9p21-deleted cells from arresting in the S-G2 phase and repairing the DNA damage induced by the treatments, thus leading to mitotic catastrophe. In addition, the downregulation of genes involved in the G2/M checkpoint induced by AG-270/MRTX1719 could further increase the efficacy of ATR inhibitors.

The translatability of these findings builds on a recent Phase I trial with AG-270 as monotherapy, which confirmed effective inhibition of MAT2A and subsequent suppression of PRMT5 activity but demonstrated only modest anti-tumor activity^42^. Similarly, early clinical reports on MRTX1719 as monotherapy showed partial responses across multiple tumor types^7^. Notably, both AG-270 and MRTX1719 were well tolerated, with reduced side effects observed in treated patients. Our findings support the use of AG-270 (or more potent MAT2A inhibitors) and MRTX1719 in combination with cytarabine and VX970 to enhance therapeutic efficacy.

Remarkably, we extended our findings to *MTAP*-deficient mesothelioma and pancreatic adenocarcinoma models, confirming the enhanced sensitivity of these cells to the combinations of MRTX1719 with cytarabine and VX970. These results underscore the broader therapeutic potential of exploiting replication stress and synthetic lethality in 9p21-deficient tumors beyond BLCA.

Finally, we obtained concordant results across two biologically distinct BLCA PDOs, supporting the robustness and relevance of the drug combinations for more physiologically relevant cancer models that retain key features of the parental tissue.

Although experiments with the PDOs support the consistency of the present data, additional studies with a larger panel of PDOs will be critical to offer deeper insight into the therapeutic potential of these combination strategies. Nonetheless, the current use of cytarabine in the clinic could increase the translation of our results into clinical testing for tumors harboring 9p21 deletions. Moreover, future work should focus on potential resistance mechanisms and toxic side effects that could arise from drug combinations.

Collectively, our findings identify nucleoside analogs as a new class of drugs with more selective activity in *MTAP*-deficient BLCA and provide a strong preclinical rationale for combination with PRMT5, MAT2A, and ATR inhibitors to maximize efficacy and selectivity. Given the high prevalence of 9p21 loss in multiple cancer types, these combinations may offer new precision medicine opportunities across a wide spectrum of malignancies.

## Methods

### Cell lines

HT1197 (RRID:CVCL_1291) and HEK293-T (RRID:CVCL_0063) were originally obtained from ATCC, while T24 (RRID:CVCL_2728) cell line from ECACC. SW1990 (RRID:CVCL_1723) and SPC111 (RRID:CVCL_D311) cell lines were kind donations of Prof. Alessandro Provenzani and Dr. Nadia Zaffaroni laboratories, respectively. SW1990 and SPC111 cells were authenticated by STR profiling. All cells were grown at 37 °C with 5% CO_2_. HT1197 were maintained in MEM (Gibco) supplemented with 10% FBS (Gibco), 1X MEM Non-Essential Amino Acids, 1 mM sodium pyruvate, and 1% penicillin-streptomycin (P/S). T24 were maintained in McCoy’s 5A plus 10% FBS and 1% P/S. HEK-T and SW1990 were maintained in DMEM supplemented with 10% FBS, 2 mM L-glutamine, and 1% P/S. SPC111 were maintained in RPMI1640 plus 10% FBS, 1 mM sodium pyruvate, 2 mM L-glutamine, and 1% P/S.

### Generation of 9p21 locus isogenic cell lines

To obtain 9p21-deleted clones, HT1197 and T24 cell lines were transduced with a lentiCas9-Blast vector (Addgene #52962, RRID:Addgene_52962). T24 and HT1197 transduced cells were selected with 2 or 3 µg/ml blasticidin (Invivogen) for 5 or 7 days, respectively, and then seeded at very low density in 96-well plates to isolate single cell clones. Cas9 expression was assessed by western blot, and one clone with intermediate Cas9 expression was selected for each cell line. We used GPP Web Portal (http://portals.broadinstitute.org/gpp/public/) to design different combinations of sgRNAs flanking the region of interest (*MTAP/CDKN2B* genes for the generation of *MTAP*^−/−^*CDKN2A*^−/−^/*CDKN2B*^−/−^ (3KO) clones, or *CDKN2A/2B* genes for the generation of *CDKN2A*^−/−^/*CDKN2B*^−/−^ (2KO) clones) that were cloned in pGS-sgRNA_Neo and pGS-sgRNA_Hygro plasmids (obtained from GenScript) (Supplementary Table 1). All sgRNAs were characterized for predicted off-targets using Cas-OFFinder^43^ (Supplementary Table 2). HT1197 and T24 Cas9-expressing clones were then transfected separately with one combination of two sgRNAs. Four combinations of sgRNAs were used to obtain 3KO clones, three were used to obtain 2KO clones, and one pair of non-targeting sgRNAs was used to obtain WT clones (Supplementary Table 1). HT1197 and T24 were transfected using TransIT-X2 (Mirus). Transfected cells were selected using 500 µg/ml neomycin and 200 µg/ml hygromycin, and then seeded at very low density in 96-well plates to isolate single cell clones. DNA was extracted using QuickExtract DNA Extraction Solution (Lucigen) according to manufacturer’s instructions, and then used for PCR amplification with Platinum SuperFi Green Master mix (Invitrogen), using primers spanning the deletion under the following conditions: 15 s 98 °C, 15 s 60 °C, 80 s 72 °C for 35 cycles. Three pairs of primers were used to detect WT and 3KO clones, and inversion of the deleted fragment (Supplementary Table 1). The PCR products were run on a 1.4% agarose gel to determine the genotype of the clones: no editing (WT), homozygous deletion (3KO), or locus inversion.

### Growth competition assay

HT1197 and T24 WT clones were transduced with a lentiviral vector carrying a mCherry reporter (mCherry/Neo, VectorBuilder), while 3KO clones were transduced with a lentiviral vector carrying an EGFP reporter (EGFP/Hygro, VectorBuilder). After selection with 500 µg/ml neomycin and 200 µg/ml hygromycin, clones were mixed and seeded in a 50:50 ratio. Cells were cultured for 3 weeks. Every 2–4 days, cells were trypsinized, and fluorescence was measured using TALI image-based cytometer (Life Technology) to determine the proportion of WT and 3KO clones over time.

### Cell cycle analysis

A Click-iT EdU 594 HCS assay (Thermo Fisher) coupled with DNA content analysis was performed to analyze cell cycle distribution of HT1197 and T24 (WT, 2KO, and 3KO). Cells were seeded in 96-well plate at 5000 cells/well. The day after seeding, 5 µM EdU was added, and cells were incubated for 2 h under normal cell culture conditions. Cells were fixed using 4% formaldehyde in PBS for 15 min at room temperature (RT), washed twice with PBS, and permeabilized using 0.1% Triton X-100 (Thermo Scientific) in PBS. Click reaction was performed following manufacturer’s instructions. Cells were then stained with HCS NuclearMask Blue (Thermo Fisher), and images acquired using 10× objective with ImageXpress Micro Confocal (Molecular Devices).

### sgRNAs cloning

sgRNAs sequences (Supplementary Table 1) for gene knockout were cloned into an optimized version of the lentiCRISPR_v1 plasmid, an all-in-one lentiviral vector carrying Cas9 protein and sgRNA under a U6 promoter with a puromycin selection marker (Cereseto’s laboratory, University of Trento). sgRNA oligonucleotides were annealed using an equal ratio of each 100 µM oligo in 10X T4 Ligase buffer (NEB). Hybridization was performed using the following thermocycling conditions: 2′ 95 °C, 2 °C/s 85 °C to 25 °C, 0.1 °C/s 25 °C to 4 °C. LentiCRISPR_v1 plasmid was digested with BsmBI-v2 enzyme in 1X NEBuffer r3.1 for 1.5 h at 55 °C. After digestion, samples were loaded on a 0.8% agarose gel, and the bands containing the DNA fragments of interest were cut from the gel and then purified using NucleoSpin gel kit (Machery-Nagel) following the manufacturer’s protocol.

Ligation of the digested plasmids with sgRNAs was performed in a 20 µl volume with 1 µl of T4 DNA ligase, 50 ng of digested plasmid, and 1 µl of hybridized sgRNAs (diluted 1:500). The ligation mix was incubated at 25 °C for 30 min and then used to transform Stbl3 competent cells. Ligation was confirmed by sequencing of the region close-by the insertion site.

### Lentiviral vector production and transduction

HEK293-T cells were seeded in 100 mm Petri dishes at 70% confluence. The day after seeding, the cell culture medium was replaced with 4 ml of DMEM (Gibco) plus 10% FBS and 2mM L-glutamine without antibiotics. In one tube, 5.3 µg of the plasmid of interest, 2.66 µg of the envelope plasmid (pVSVg), and 4 µg of the packaging plasmid (psPax2) were mixed, and 31 µl of CaCl_2_ 2 M were added to the solution. After gentle mixing, the solution was added to 250 µl of 2X Hepes buffered saline (HBS), mixed, and incubated for 20 min at RT. The transfection solution was then gently added to the cells, and HEK293-T was incubated overnight. The day after, the cell culture medium with the transfection mix was replaced with fresh medium. Supernatant containing viral vectors were collected at 48 h and 72 h post-transfection, centrifuged at 3000 rmp and filtered using 0.45 µm filters to remove cell debris. Finally, the viral vectors were directly used for transduction or stored at −80 °C. The day before transduction, the cell line of interest was seeded at 70% confluence in a 6-well plate. Cells were transduced by spinoculation (30 min, 2500 rpm at 30 °C) with 2–8 µg/ml polybrene (Santa Cruz Biotechnology) according to the cell line used and incubated overnight. Cells were then trypsinized and selected with 2–3 µg/ml puromycin (InvivoGen).

### RT-qPCR

RNA was extracted from cells using RNeasy Mini Kit (QIAGEN) following manufacturer’s instructions and quantified by Nanodrop (Thermo Scientific). 1 µg of RNA was converted to cDNA using RevertAid First Strand cDNA Synthesis Kit (Thermo Fisher). qRT-PCR was performed in triplicate using KAPA SYBR FAST qPCR kit (Biosystem KK4602) in 96- or 384-well PCR plates (Biorad).

The 2^−ΔΔCt^ method was used to analyze the relative changes in gene expression. Data were normalized to GAPDH expression, and the fold change in gene expression was calculated relative to cells transduced with non-targeting sgRNA. List of the used primers is reported in Supplementary Table 1.

### Immunoblotting

Cells were detached on ice using a cell scraper, washed with ice-cold PBS 1X, and lysed with ice-cold RIPA buffer supplemented with protease and phosphate inhibitors. Cell pellets were incubated on ice for 30 min with occasional vortexing. After lysis, samples were centrifuged at 13,000 rpm for 15 min at 4 °C, and supernatants were collected. Protein concentration of each sample was determined using a Pierce BCA protein assay kit (Thermo Fisher) with 5 µl of protein lysate. After 30 min incubation at 37 °C in the dark, the absorbance at 562 nm was measured with Varioskan LUX multimode microplate reader (Thermo Scientific) and protein concentrations determined by comparison to a standard curve.

In order to prepare samples for gel electrophoresis, 6X sample buffer was added, and samples were denatured in reducing conditions at 95 °C for 10 min before loading onto 4-15% Mini-PROTEAN TGX Stain-Free gels (Biorad). Gels were run at 90–120 V using Tris/Glycine Buffer (Biorad). Proteins were transferred from the gels to a PVDF membrane using the semi-dry transfer technology Trans-Blot Turbo Transfer System (Biorad), with the following protocol: 1.3 A for 7 min.

Membranes were blocked with 5% milk in TBS-T 1X buffer for 1 h at RT. Primary antibodies (Supplementary Table 3) were diluted in blocking buffer (either 5% milk or BSA) and incubated overnight at 4 °C on a rocking platform. Membranes were then washed three times for 10 min with 1X TBS-T, and incubated with the appropriate secondary antibody for 1 h at RT, followed by three 10 min-washes with 1X TBS-T. The chemiluminescent signal was detected with Biorad Chemidoc Imaging Systems using Amersham ECL Prime or Select reagents (GE Healthcare Life Sciences).

### Cell lines immunofluorescence

γH2AX staining was evaluated in BLCA cells treated with the drugs of interest for 5 (T24) or 7 (HT1197) days. HT1197 clones (800 cells/well) and T24 clones (400 cells/well) were seeded in CellCarrier 96 Ultra plates (PerkinElmer), and drugs were added the following day. At the end of treatment, cells were fixed with 4% PFA in PBS for 15 min at RT, and permeabilized using a solution containing 3% BSA, 0.3% Triton X-100 in PBS for 45 min at RT. Anti-γH2AX primary antibody (1:1000, 2577 Cell Signaling Technology) was then added to the wells and incubated for 1 h at RT. After washing with 200 µl 3% BSA in PBS (3 times, 5 min each), anti-rabbit Alexa Fluor 488 secondary antibody (1:500, Invitrogen) was added, and incubated for 1 h at RT. Wells were then washed with 3% BSA in PBS (3 times, 5 min each), and nuclei were stained with Hoechst 33342 (Thermo Fisher), incubated for 10 min. Multiple fields within a well were acquired using a 20× objective with ImageXpress Micro Confocal (Molecular Devices) to include a minimum of 300 cells per condition. Objects with a minimum nuclei area of 100 μm^2^ were selected, and the proportion of cells with >5 foci per nucleus was calculated. For micronuclei quantification, 300 nuclei were assessed per experimental condition.

### Annexin V apoptosis assay

T24 (8 × 10^4^) and HT1197 (1 × 10^5^) cells were seeded in 100 mm Petri dish and let adhere overnight. The day after seeding, cells were treated with AG-270 or MRTX1719. After 48 h, drugs were combined with cytarabine or VX970 for different amounts of time according to the cell line and treatment administered. After treatments, both attached and floating cells were collected, washed with PBS, counted, and resuspended in 1X Binding Buffer (BD Pharmingen) at a concentration of 3 × 10^6^ cells/ml. 100 µl of cell suspension (3 × 10^5^) were incubated with 5 µl of APC Annexin V (BD Pharmingen) for 15 min at RT in the dark and then resuspended in 400 µl of 1X Binding Buffer for flow cytometry analysis. 5 µl of Propidium Iodide (PI, Invitrogen) were added to each tube just before measurement. For each sample, 10,000 events were acquired using BD FACSymphony™ A1 Cell Analyzer, and FlowJo™ Software was used to analyze the data. Unstained cells, cells stained with APC Annexin V only, or PI only, were used to set up compensation and quadrants.

### Cell viability assays

Cell viability was determined by Cell Counting kit-8 (CCK-8, Dojindo) and crystal violet assays. Cells were seeded at optimized densities in 24- or 48-well plates and let to attach overnight before being treated with the indicated compounds (Supplementary Table 4). Medium was substituted every 2–3 days. At the end of treatment, CCK-8 diluted in cell culture medium (1:40) was added to the cells and incubated for 1–2 h at 37 °C. Absorbance at 450 nm was then measured with Varioskan LUX multimode microplate reader (Thermo Scientific). CCK-8 solution was removed, cells were fixed with 4% formaldehyde, and stained with crystal violet (0.1% crystal violet, 20% EtOH, and H_2_O) for 30 min at RT. Plates were rinsed thoroughly and then allowed to dry overnight before pictures were taken. For quantification, crystal violet was dissolved with 10% acetic acid, and the absorbance was measured at 595 nm with Varioskan LUX multimode microplate reader. Cell viability was calculated by normalizing the values to DMSO-treated wells.

### Multiparametric drug screening

A multiparametric drug screening was performed with an HT1197 WT clone and an HT1197 3KO clone. Cells were seeded in 384-well plates with a Freedom EVO platform (Tecan), using a 96-channel pipetting head. Cells were let adhere overnight and then treated with a library of 2349 compounds, tested in duplicate at 1 µM. The tested drugs belong to two collections: (a) Anti-cancer compounds (Selleck): 349 anti-cancer small molecules in clinical use or in clinical trials; (b) Spectrum Collection (MicroSource): 958 known drugs that have been used in human therapy; 629 natural products, and derivatives with undetermined biological activities; 343 compounds with reported biological activity at the experimental level; and 70 compounds approved for, and restricted to, agricultural use. After 48 h of treatment, cells were fixed with 4% PFA and stained with Hoechst and Alexa Fluor 555 phalloidin using the plate washer-dispenser EL406 (BioTek). Fluorescence images were acquired using Operetta High Content Imaging System (Revvity) at 10x magnification in widefield mode, two fields per well, and analyzed using CellProfiler^44^. For each object (i.e., cell), 12 phenotypic features characterizing area/shape and intensity were analyzed at the nuclear- and cell-level (i.e., area, compactness, form factor, perimeter, DNA intensity, phalloidin intensity). To analyze the activity of the drugs on viability (i.e., cell number were obtained by nuclei count) and morphological features, we first calculated z-scores using the following formula: (X_S_−X_V_)/SD_V_; where X_S_ is the mean signal from technical replicates for each drug treatment, X_V_ is the mean of technical replicates of the vehicle, and SD_V_ the standard deviation of the technical replicates of the vehicle (Supplementary Tables 5 and 6). To prioritize the drugs that were selectively effective in 3KO cells, we selected the drugs that reduced cell viability in the 3KO clone (z-score ≤ −2.5) and not in the WT clone (−1.5 ≤ z-score ≤ 1.5). In terms of morphology, we defined as significantly morphologically altered the drugs that scored within the interquartile range (IQR) for WT cells and outliers for 3KO cells, here defined as below Q_1_−1.5 × IQR or above Q_3_−1.5 × IQR (Supplementary Table 7).

### Correlation analysis

To identify drugs that induce morphological alterations similar to those caused by cytarabine, we calculated the Pearson correlation coefficient between cytarabine-induced morphological features and those induced by other drugs in the screening (Supplementary Table 8).

### Clinical details of patient-derived organoid lines

PDO lines, TN-001 and BlCa197, were generated from specimens obtained from patients who underwent cystectomy at the Inselspital, University Hospital, in Bern, or Spitalzentrum Biel, respectively. Specifically, BlCa197 cells were isolated from a 54-year-old female patient with high-grade MIBC with mixed urothelial and sarcomatoid histology. This patient underwent neoadjuvant cisplatin/gemcitabine treatment followed by radical cystectomy. TN-001 was obtained from a 53-year-old male patient diagnosed with high-grade MIBC with mixed urothelial and sarcomatoid histology. This patient received MVAC combination followed by radical cystectomy. The research has been performed in accordance with the Declaration of Helsinki. Patients included in this study provided written informed consent (Cantonal Ethical approval KEK 06/03 and 2017-02295), and all data were treated according to the protocol. The research project does not include study specific intervention.

### Patient-derived organoids (PDOs) establishment

Tissues were mechanically disrupted with a scalpel, washed with Basis medium (Advanced DMEM/F-12 (Gibco) containing 2 mM GlutaMAX (Gibco), 10 mM HEPES (Gibco), and 100 µg/ml Primocin (InvivoGen)), and digested with an enzyme mix made of 5 mg/ml collagenase II (Thermo Fisher 17101015) dissolved in Basis medium, 15 µg/ml DNase I (Roche), and 10 µM Y-27632 ROCK inhibitor (Selleck). Tissue was incubated at 37 °C for 1 h, mixing every 10 min. After digestion, the tissue was washed with Basis medium, and the cell pellet was incubated at 37 °C for 10 min with 2-5 ml of TrypLE Express (Thermo Fisher) according to the cell pellet amount. Cell suspension was filtered through a 70 µm cell strainer and was extensively washed with Basis medium. Cells were centrifuged and resuspended in Matrigel (Corning) at a density of 5 × 10^3^ cells/well in 6-well plates. After Matrigel had solidified, 2 ml of organoid medium were added, and cells were incubated at 37 °C with 5% CO_2_. Organoid medium was substituted every 2–3 days. The organoid medium contained: Advanced DMEM/F-12 (Gibco), supplemented with 5% FBS (Gibco), 2 mM GlutaMAX (Gibco), 10 mM HEPES (Gibco), Primocin 100 µg/ml (InvivoGen), Fungin 10 µg/ml (InvivoGen), 10 μM Y-27632 (Selleck), and 10 ng/ml Mouse EGF Recombinant Protein (Gibco).

To obtain TN-001 PDOs with doxycycline-inducible MTAP overexpression, the *MTAP* gene was cloned into pCW57.1 (Addgene #41393), which was then used to transduce TN-001 cells. For *MTAP* knockout in BlCa197 PDOs, *MTAP* sgRNA sequence was cloned into a lentiCRISPR_v1 plasmid as previously described, which was then used to transduce BlCa197 cells.

### PDOs drug screen

PDOs were dissociated into single cells with TrypLE Express at 37 °C for 10 min. Cells were then counted, resuspended in Matrigel (Corning), and seeded in a white 96-multiwell plate (Revvity) (2000 cells per 10 μl Matrigel per well). After Matrigel solidified, 200 µl of organoid medium were added. Three days post-seeding, the medium was replaced with organoid medium with different drug concentrations (2 technical replicates per condition). Medium was substituted every 2–3 days. At the end of treatment, cell viability was assessed using CellTiter-Glo 3D (Promega) (1:3 with culture medium) according to the manufacturer’s protocol. Luminescence was measured using Varioskan LUX multimode microplate reader (Thermo Scientific).

### FFPE multiplex immunofluorescence

After tissue/PDOs processing and embedding, 4μm tissue sections were obtained for hematoxylin, eosin (H&E), and immunofluorescence staining. Briefly, antigen retrieval was performed in citrate buffer for 10 min, followed by blocking with 10% Donkey serum/PBS/0.05% Tween for 2 h at room temperature. Human primary antibodies (Supplementary Table 3) were diluted 1:200 in 10% Donkey serum/PBS/0.05% Tween, and incubated at 4 °C overnight. Fluorescent secondary antibodies, Alexa Fluor 555 donkey anti-mouse (A-31570), Alexa Fluor 488 donkey anti-rabbit (A-21206), and Alexa Fluor 647 donkey anti-goat (A-21447), were diluted 1:250 in 10% Donkey serum/PBS/0.05% Tween and incubated for 90 min at room temperature. Antibody multiplexing was planned according to the primary host species. 4′6-diamidino-2-phenylindole (DAPI) dye (1:500) was added immediately after secondary antibody incubation for nuclei counterstaining. Afterwards, ProLong™ Diamond Antifade Mountant (Thermo Fisher Scientific) was used for mounting the slides and keeping the fluorescent staining stable.

Brightfield and IF images were acquired with a slide scanner (3DHistech Pannoramic 250 Flash II) at 20× magnification. Subsequently, all images were processed with 3D Histech case viewer software.

## Supplementary information

Supplementary Information

Supplementary Tables

## Data Availability

Multiparametric drug screening raw data have been deposited in Zenodo 10.5281/zenodo.18957959. Additional data generated in this study are available upon request from the corresponding authors.

## Code availability

The underlying code for this study to pre-process drug screening image data is not publicly available but may be made available to qualified researchers on reasonable request from the corresponding authors.
