# Supplementary material for: Therapeutic vulnerabilities exposed by the 9p21 loss identified through multiparametric drug screening inform rational combination strategies
Source: NPJ Precis Oncol. 2026 Apr 18;10:232. doi: 10.1038/s41698-026-01434-w (PMC13276166; doi:10.1038/s41698-026-01434-w)

Supplementary Figure1

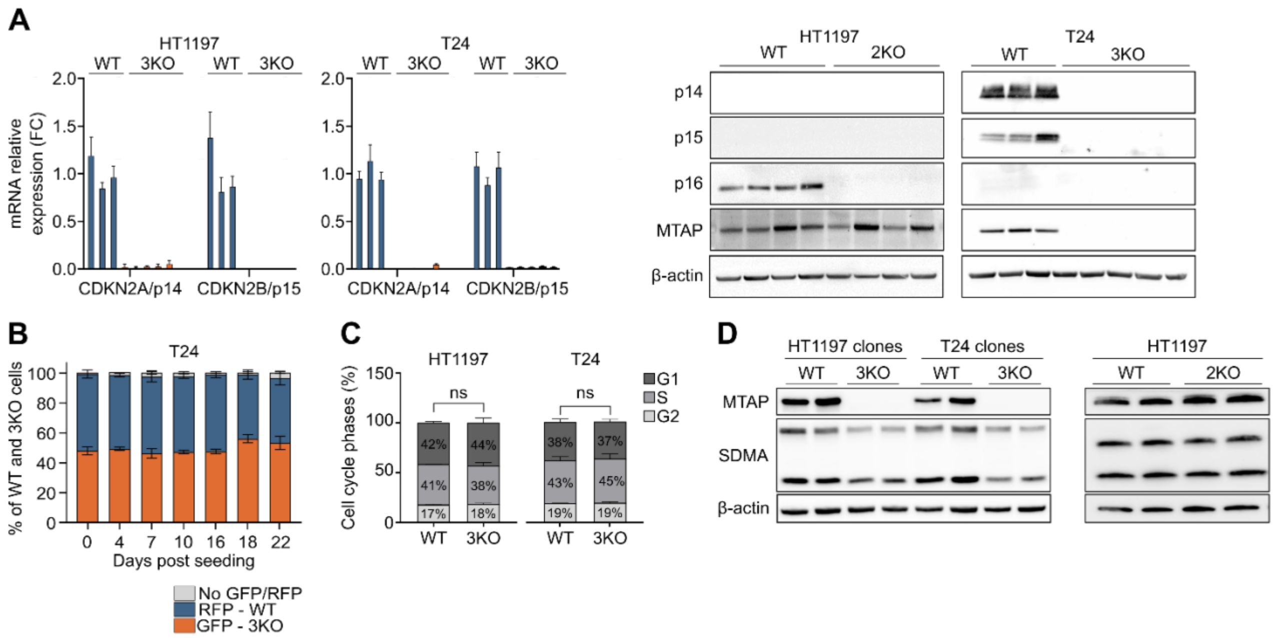

E

Drug name	Library	WT cell count	3KO cell count	Target
3-hydroxy-3',4'-dimethoxyflavone	MicroSource	-1.3047	-3.0170	Unknown
4-naphthalimidobutyric acid	MicroSource	-1.2957	-2.5270	Unknown
abitrexate (methotrexate)	Selleck	-1.2101	-2.8319	DHFR
aminacrine	MicroSource	-0.7563	-3.5973	Unknown
aristolochic acid	MicroSource	-1.3938	-2.7587	NF-κB
asiatic acid	MicroSource	-0.6550	-2.8131	p38 MAPK
at9283	Selleck	-1.3016	-2.6825	JAK2/3
bosutinib (ski-606)	Selleck	-1.4733	-2.5430	Src/Abl
carbenoxolone sodium	MicroSource	-0.2319	-2.7262	11β-HSD
ci-1040(pd184352)	Selleck	-1.3413	-2.6087	MEK1/2
cytarabine	Selleck	-0.3088	-2.6870	DNA/RNA Synthesis
erysolin	MicroSource	-1.1096	-3.0762	CYP1A
hinokitiol	MicroSource	-1.4121	-2.7852	DNA Methyltransferase
nu7441 (ku-57788)	Selleck	-1.2276	-3.3739	DNA-PK
pteryxin	MicroSource	-0.8669	-2.7391	BChE
rhizocarpic acid	MicroSource	-1.1986	-2.6183	Unknown
sns-314 mesylate	Selleck	-1.3544	-2.8377	Aurora Kinase
thermopsine perchlorate	MicroSource	-1.3220	-2.5350	Unknown

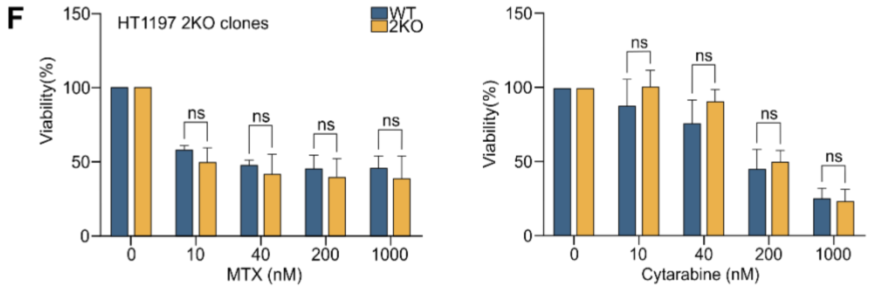

Supplementary Figure 1: Characterization of the HT1197 and T24 isolated clones, and activity of cytarabine and methotrexate in HT1197 and T24 2KO models.
A. Left, *CDKN2A*/p14 and of *CDKN2B*/p15 expression measured by RT-qPCR in HT1197 and T24 WT and 3KO clones. Right, western blot analysis of HT1197 and T24 WT, 2KO ($CDKN2A^{-/-}/2B^{-/-}$), and 3KO ($CDKN2A^{-/-}/2B^{-/-}/MTAP^{-/-}$) clones with the indicated antibodies. **B.** Growth competition assay for T24 9p21 isogenic pair. WT (RFP-positive) and 3KO (GFP-positive) cells were co-cultured for 3 weeks, and the proportion of each cell population was measured every 3-4 days using Tali image-based cytometer (mean ± SD, n = 3). 3KO cells are more abundant from day 18 (P < 0.01, unpaired t-test). **C.** Cell cycle analysis of the isolated HT1197 and T24 clones measured by EdU incorporation (2h incubation) coupled with DNA content analysis. The plotted values represent the mean ± SD of the analysis of 3 WT and 5 2KO/3KO clones for the two cell lines. Each experiment was performed in n = 2 biological replicates. P-values were calculated using two-sided Wilcoxon test. **D.** Western blot analysis of SDMA levels in HT1197 and T24 WT, 2KO and 3KO clones. **E.** List of the 18 drugs that showed 3KO selective viability reduction (i.e., -1.5 ≤ WT z-score ≤ 1.5; 3KO z-score ≤ -2.5). The molecular target of each drug is reported if either present in Selleckchem website, DrugBank database or MedChemExpress database. **F.** Crystal violet survival assays performed with 2 HT1197 WT and 2 HT1197 2KO clones (mean ± SD, n = 6, 3 biological replicates per clone). Cells were treated with cytarabine and MTX for 7 days. P-values were calculated by unpaired t-test. *P < 0.05; **P < 0.01

Supplementary Figure2

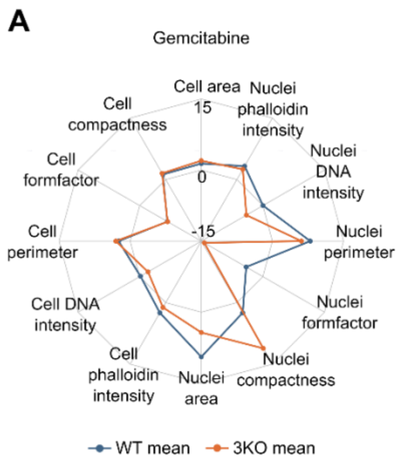

Supplementary Figure 2: Morphological alterations induced by gemcitabine in WT and 3KO clones. **A.** Radar plots showing the phenotypic alterations induced by gemcitabine in HT1197 WT and 3KO cells.

Supplementary Figure 3

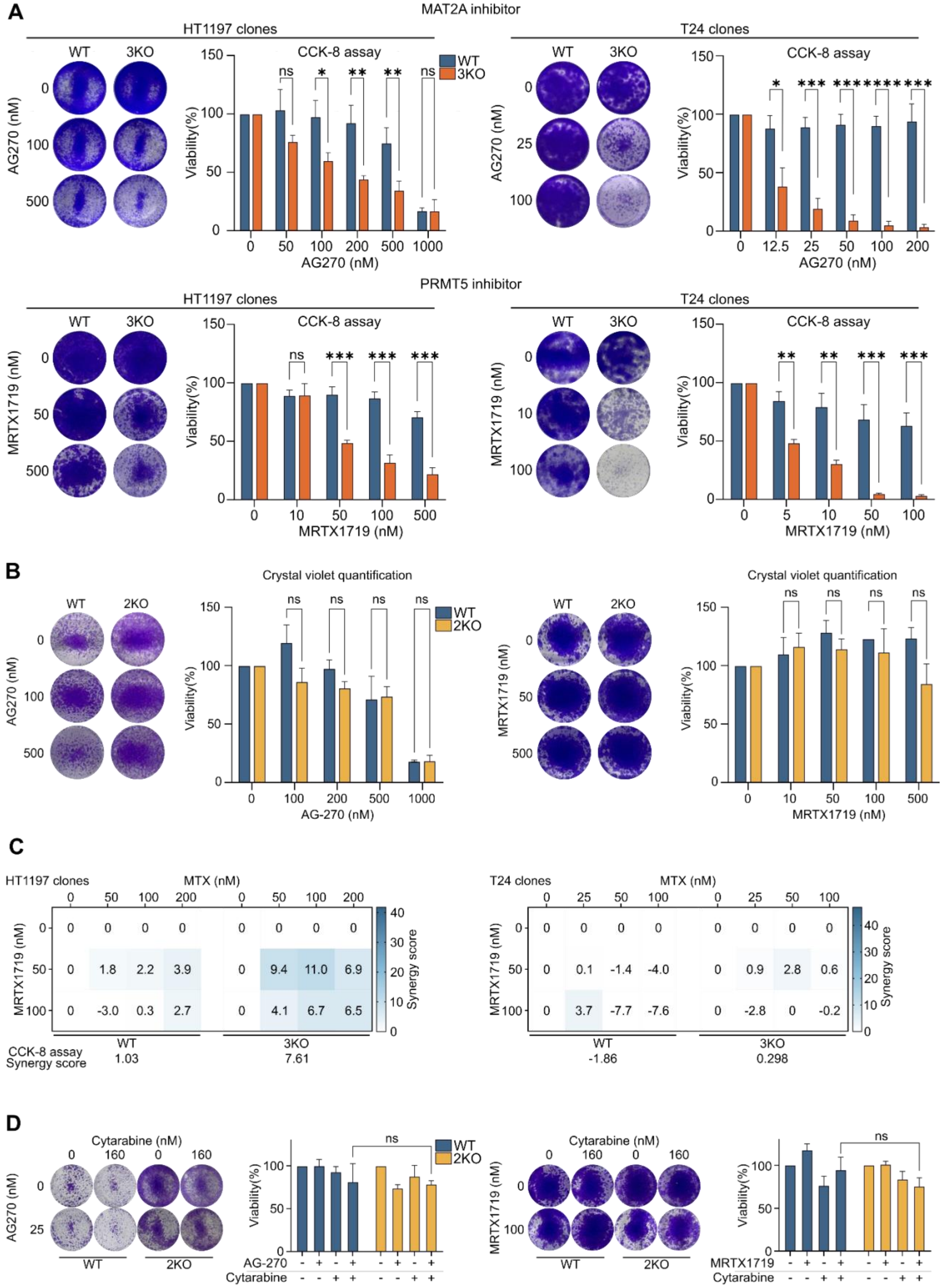

Supplementary Figure 3: Activity of AG-270, MRTX1719 and drug combinations in HT1197 and T24 WT, 2KO and 3KO clones.

A. Cell viability assays (crystal violet assay, left and Cell Counting Kit-8 (CCK8) assay, right) in HT1197 and T24 WT and 3KO clones treated with the MAT2A inhibitor AG-270 (10 and 7 days treatment, respectively) and the PRMT5 inhibitor MRTX1719 (7 and 5 days treatment, respectively) (mean ± SD, n = 3). P-values were calculated by multiple unpaired t-test. **B.** Crystal violet assay quantification of AG-270 and MRTX1719 treatments in HT1197 WT and 2KO cells (mean ± SD, n = 3). P-values were calculated by unpaired t-test. **C.** Dose-response matrixes of MTX - MRTX1719 combinations in HT1197 (left) and T24 (right) WT and 3KO clones. HT1197 and T24 clones were treated with MRTX1719 for 2 days followed by combined MRTX1719 and MTX treatment for 3 (T24 clones) or 5 (HT1197 clones) days. Cell viability was measured by CCK-8 assays. HSA synergy was calculated using SynergyFinder 3.1 and HSA synergy scores and summary synergy scores with 95% confidence interval for the CCK-8 experiments are shown. **D.** Cytarabine - AG-270 and cytarabine - MRTX1719 combinations in HT1197 WT and 2KO clones. Cells were treated for 2 days with AG-270 or MRTX1719 alone followed by combined AG-270 and cytarabine treatment for 5 days. Cell viability was measured by crystal violet and CCK-8 assays. Crystal violet experiments and barplots show representative images of selected cytarabine and AG-270 combinations, and of CCK-8 measured cell viability, respectively. P-values were calculated by unpaired t-test. *P < 0.05; **P < 0.01; ***P < 0.001; ****P < 0.0001

Supplementary Figure4

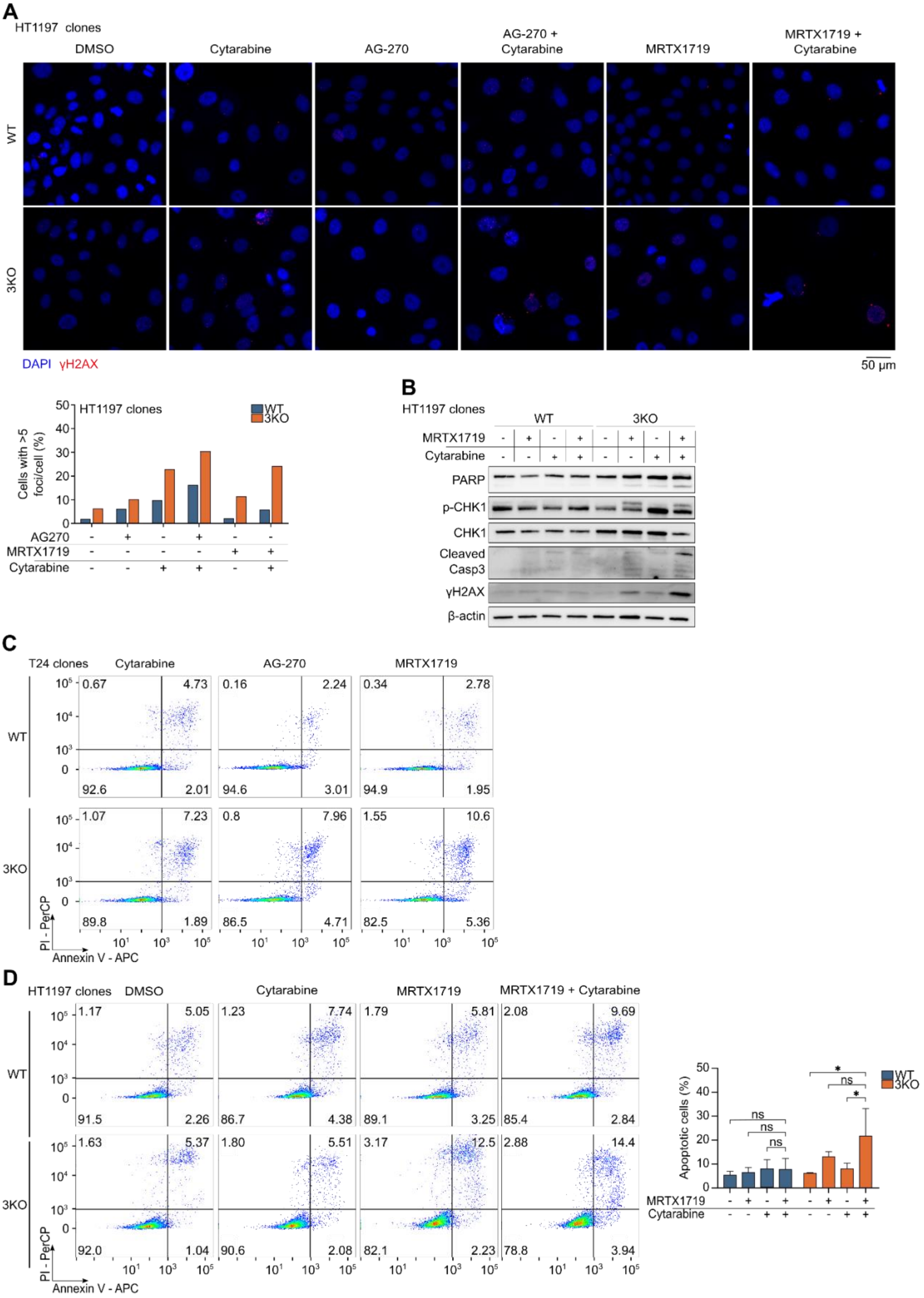

Supplementary Figure 4: Biological effects of cytarabine and its combination with MRTX1719 in HT1197 WT and 3KO clones.

A. Representative images and quantification of γH2AX foci in HT1197 WT and 3KO cells treated with the indicated drugs. Data represent the proportion of cells with >5 foci/nucleus. **B.** Western blot analysis of the indicated proteins in HT1197 WT and 3KO cells treated with DMSO (as control) or MRTX1719. HT1197 cells were treated with MRTX1719 (50nM) for 2 days, followed by combination with cytarabine (160nM) for 5 days. **C.** Representative FACS profiles of Annexin V - PI staining for T24 9p21 isogenic pair treated with the indicated drugs (mean ± SD, n = 3). T24 cells were treated with AG-270 (25nM), MRTX1719 (10nM) for 5 days or cytarabine (200nM) for 3 days. **D.** Representative FACS profiles (left) and quantification (right) of Annexin V - PI staining for HT1197 WT and 3KO cells treated with the indicated drugs, alone or in combinations (mean ± SD, n = 3). HT1197 cells were treated with DMSO (as control) or MRTX1719 for 2 days, followed by combination with cytarabine for 5 days. P-values were calculated by one-way ANOVA. *P < 0.05

Supplementary Figure5

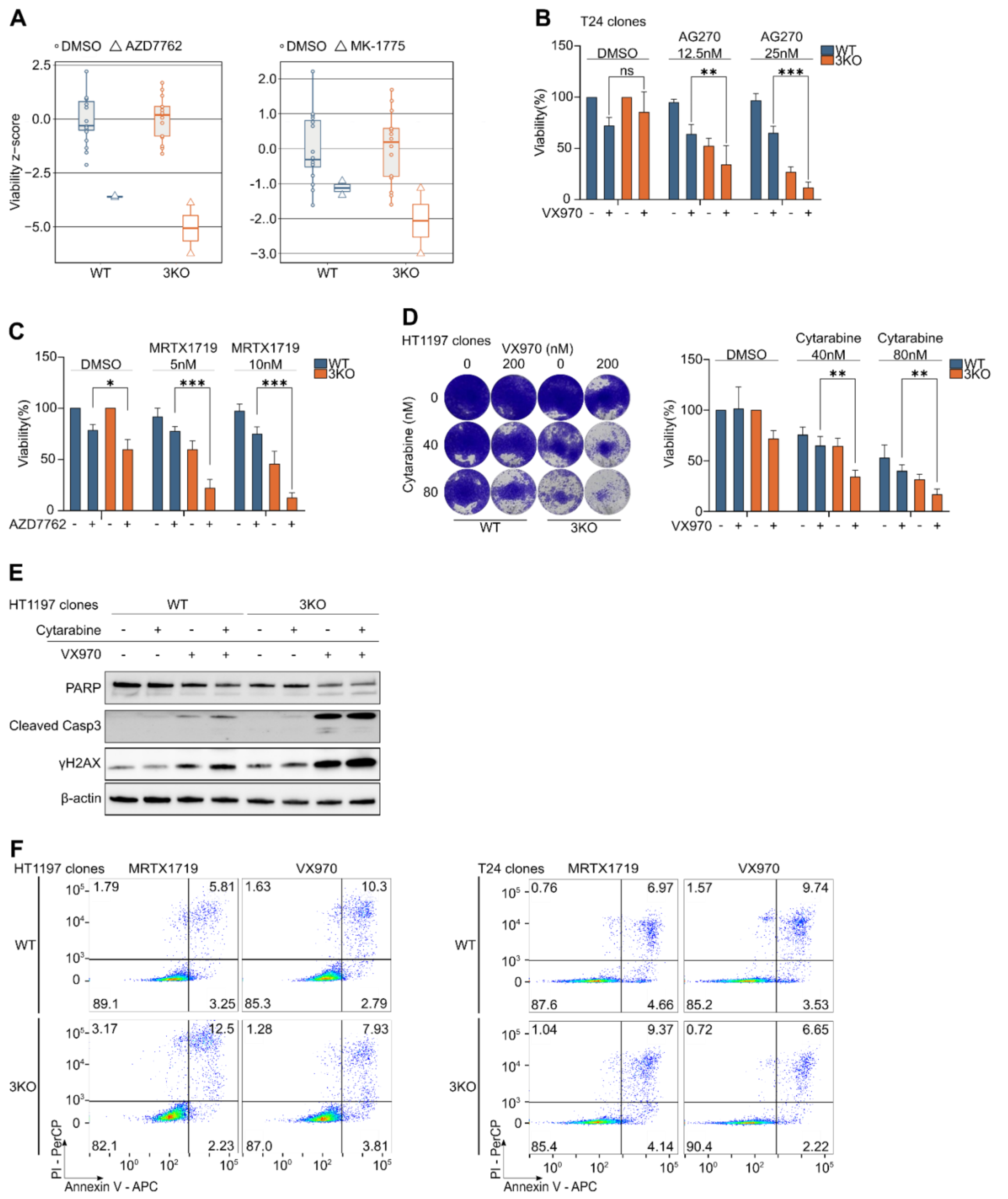

Supplementary Figure 5: The ATR inhibitor VX970 combines with AG-270 and AZD7762 in T24 cells with 9p21 loss.

A. Drug screening results of the impact of CHK1 (AZD7762) and WEE1 (MK-1775) inhibitors on cellular viability in the HT1197 9p21 isogenic pair. **B.** CCK-8 assays of T24 cells treated with AG-270 in combination with VX970 (mean ± SD, n = 3). T24 cells were treated with DMSO (as control), or AG-270 for 2 days, followed by combination with VX970 for 4 days. P-values were calculated by

unpaired t-test. **C.** CCK-8 assays of T24 cells treated with MRTX1719 in combination with AZD7762 (n = 3). T24 cells were treated with DMSO (as control), or MRTX1719 for 2 days, followed by combination with AZD7762 for 3 days. **D.** Crystal violet survival assays and CCK-8 assays of HT1197 cells treated with cytarabine in combination with VX970 (mean ± SD, n = 3). HT1197 cells were treated with either DMSO (as control) or cytarabine for 2 days followed by combined treatment with VX970 for 5 more days. Cell viability was measured by crystal violet and CCK-8 assays. **E.** Western blot analysis of the indicated proteins in HT1197 WT and 3KO cells treated with DMSO, cytarabine or VX970 as described in D. **F.** Representative FACS profiles of Annexin V - PI staining for HT1197 and T24 9p21 isogenic pairs treated with the indicated drugs (mean ± SD, n = 3). HT1197 cells were treated with MRTX1719 (50nM) for 7 days or VX970 (200nM) for 5 days. T24 cells were treated with MRTX1719 (10nM) for 6 days or VX970 (200nM) for 4 days. *P < 0.05; **P < 0.01; ***P < 0.001

Supplementary Figure6

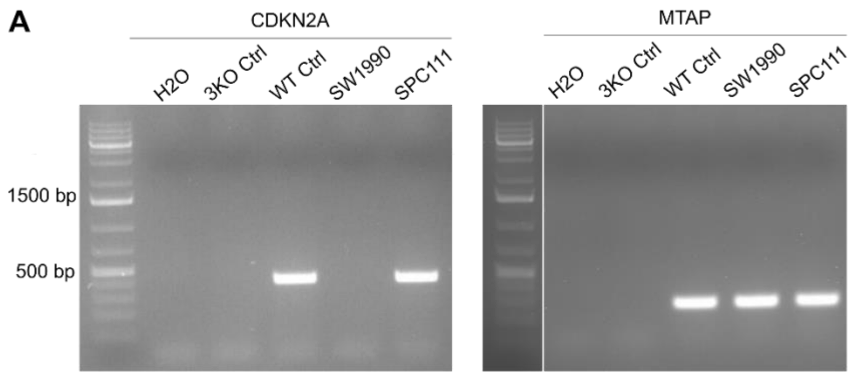

Supplementary Figure 6: Characterization of SW1990 and SPC111 cell lines.
A. Agarose gel electrophoresis of PCR products obtained using primers for CDKN2A and MTAP genes. DNA marker with sizes of DNA fragments is indicated on the left. DNA from WT and 3KO clones served as positive and negative controls, respectively.

Supplementary Figure7

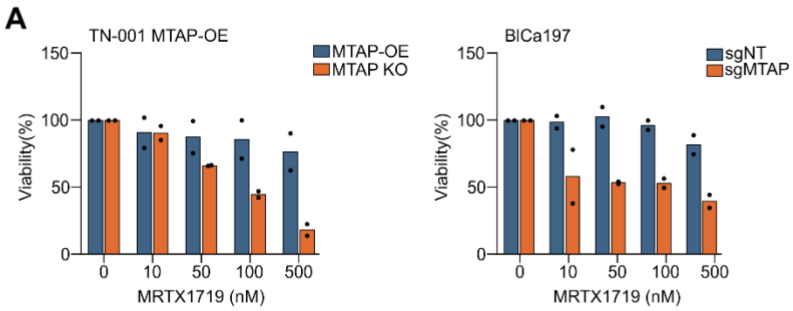

Supplementary Figure 7: Activity of MRTX1719 in human BLCA PDOs edited for MTAP expression.
A. Cell viability assays (CellTiter-Glo 3D) of TN-001 MTAP overexpressing (OE) and BlCa197 (WT-sgNT and MTAP KO-/sgMTAP) PDOs treated with the PRMT5 inhibitor MRTX1719 (7 days of treatment) (n = 2). TN-001 were treated with doxycycline (Dox) 1µg/ml to induce MTAP overexpression or ethanol (EtOH) as control.

List of Supplementary Tables

Supplementary Table 1: Study oligo sequences

Supplementary Table 2: Number of Off Targets calculated with Cas-OFFinder for the set of guides

Supplementary Table 3: List of antibodies

Supplementary Table 4: List of compounds used for cell viability assays

Supplementary Table 5. Drug screening z-scored nuclei count

Supplementary Table 6. Drug screening phenotypic scores

Supplementary Table 7. List of drugs that induced selective morphological alterations in 3KO cells for each feature

Supplementary Table 8. Similarity of the phenotypic features induced by cytarabine and the screened drugs

Uncropped blots and gels

Figure 1B

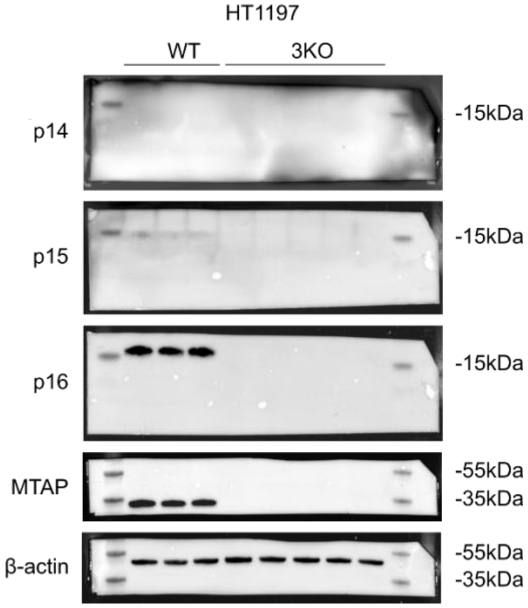

Figure 4C

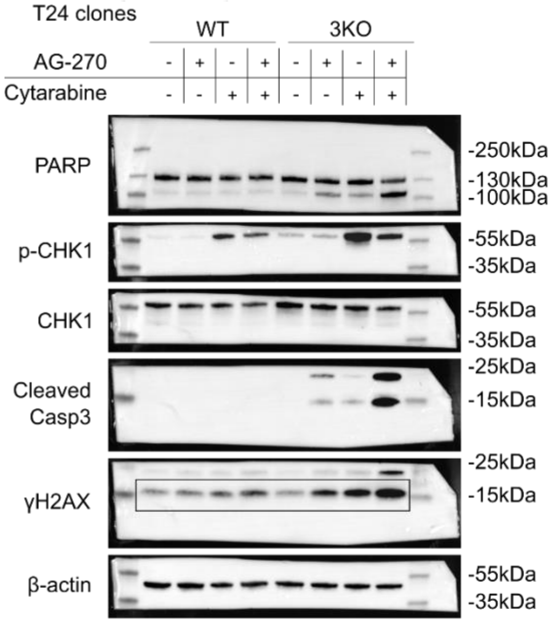

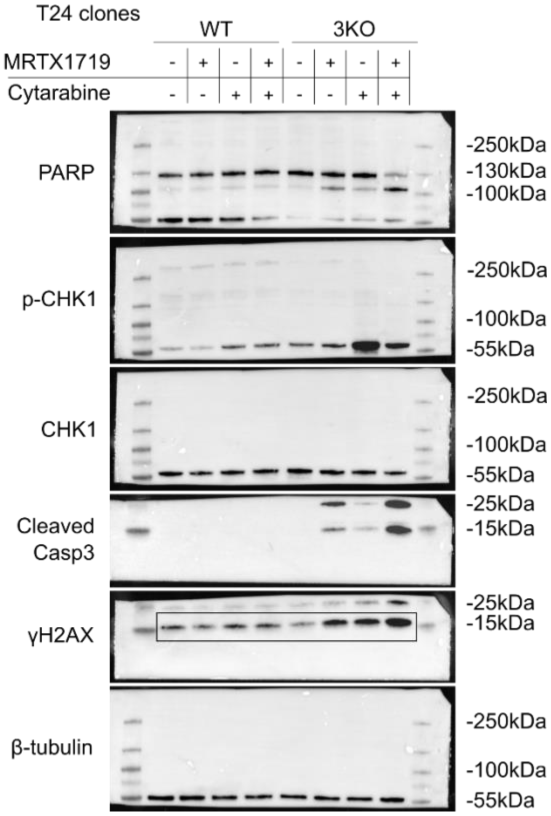

Figure 5C

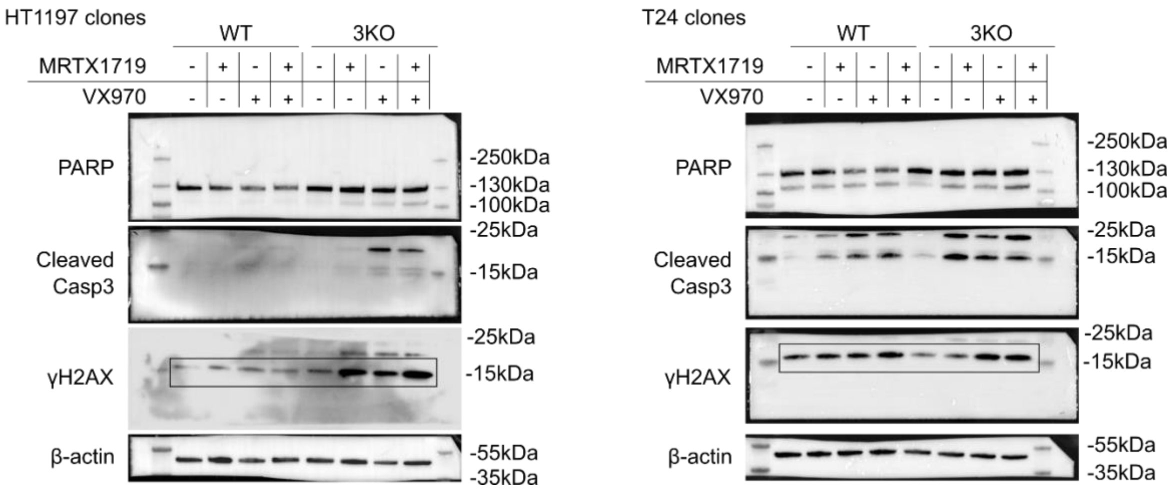

Figure 6B

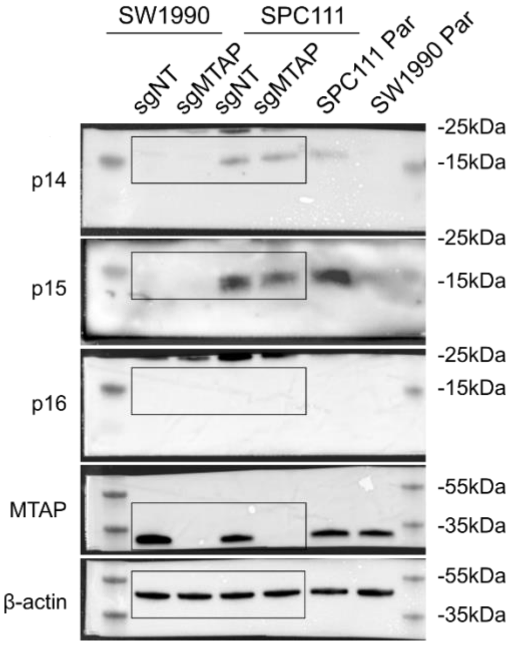

Figure 7B

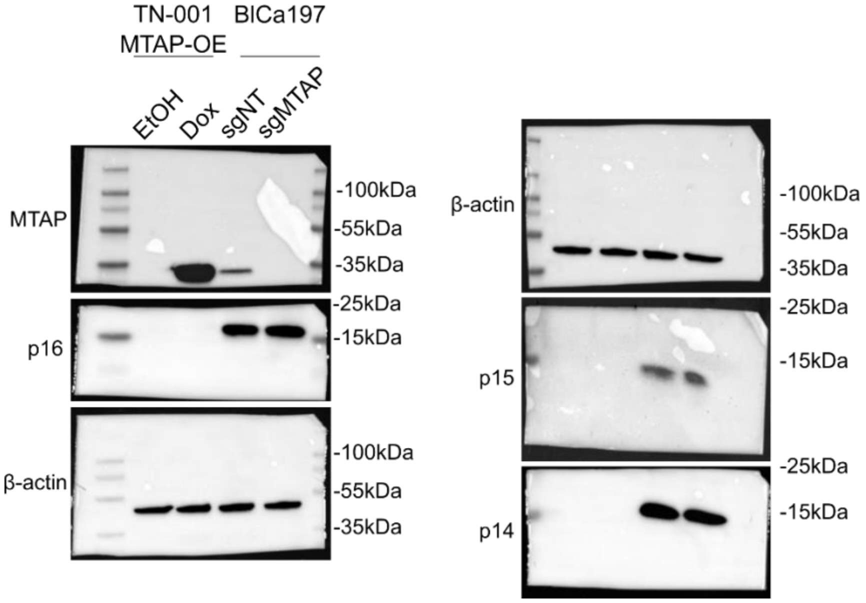

Supplementary Figure 1A

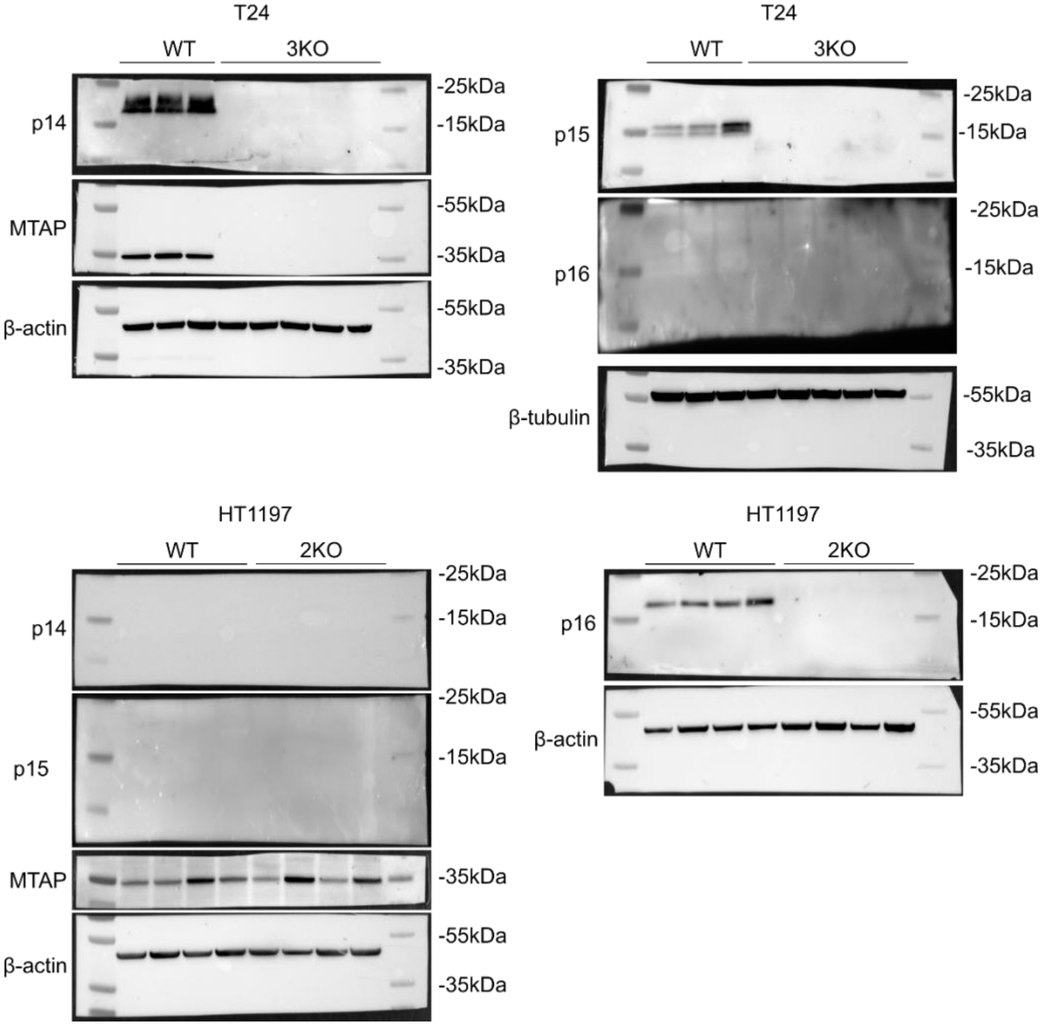

Supplementary Figure 1D

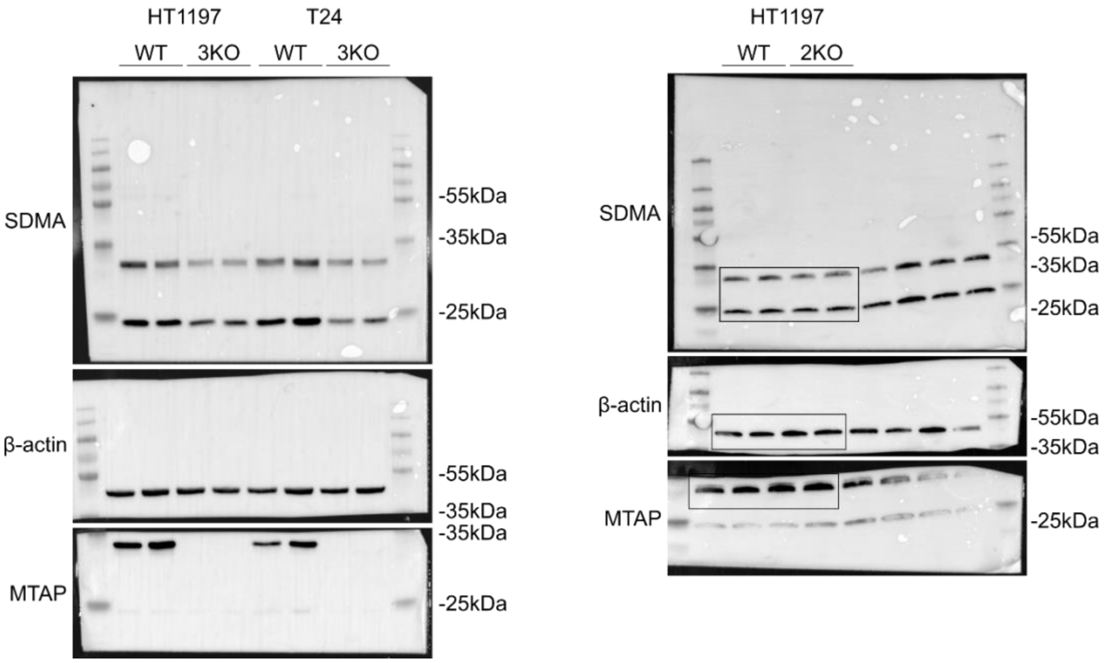

Supplementary Figure 4B

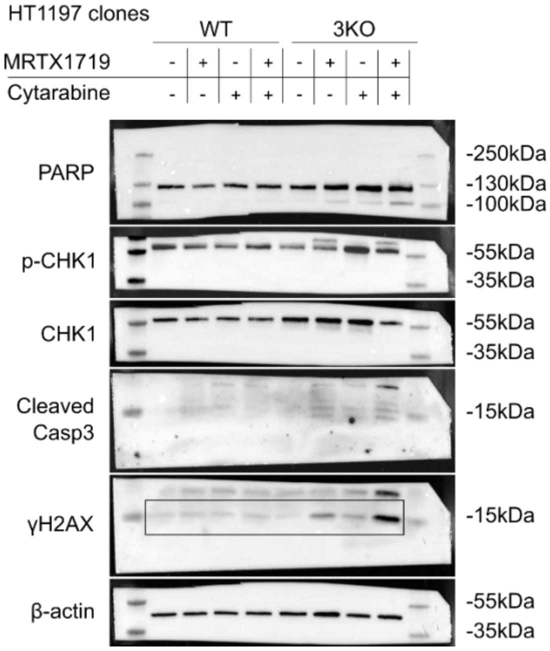

Supplementary Figure 5E

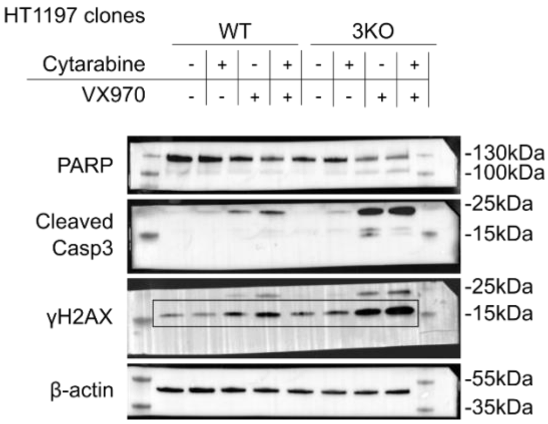

Supplementary Figure 6A

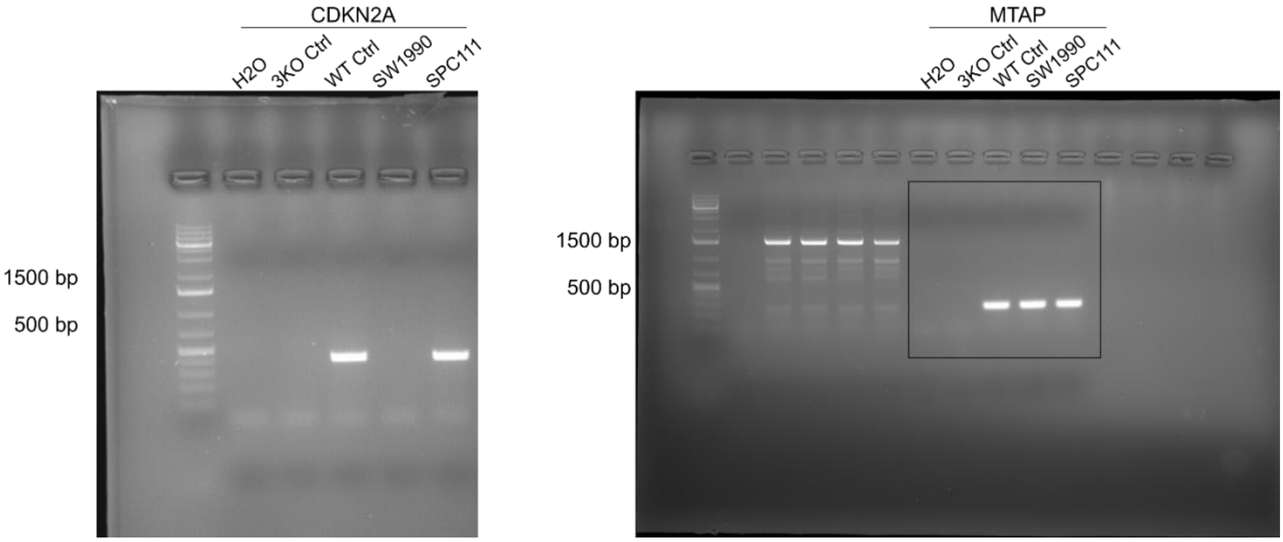

Supplement: Supplementary file 1 — Supplementary Information [file 41698_2026_1434_MOESM1_ESM.pdf]
